# Mixed‐Dimensional Floating Gate Phototransistors for Mixed‐Modal In‐Sensor Reservoir Computing

**DOI:** 10.1002/advs.202502694

**Published:** 2025-05-08

**Authors:** Weilun Ouyang, Qirui Zhang, Jiangang Chen, Xiao Luo, Xuemei Wang, Yingying Chen, Fan Yang, Qi Nie, Qing Liu, Fucai Liu

**Affiliations:** ^1^ School of Optoelectronic Science and Engineering University of Electronic Science and Technology of China No.2006, Xiyuan Ave, West Hi‐Tech Zone Chengdu Sichuan 611731 P. R. China; ^2^ State Key Laboratory of Electronic Thin Films and Integrated Devices University of Electronic Science and Technology of China No.2006, Xiyuan Ave, West Hi‐Tech Zone Chengdu Sichuan 611731 P. R. China

**Keywords:** 2D materials, neuromorphic devices, protection of endangered species, quantum dots (QDs), reservoir computing (RC)

## Abstract

Novel neuromorphic devices constructed from low‐dimensional materials have demonstrated significant potential in visual perception and information processing. Colloidal quantum dots (QDs) exhibit strong light absorption and tunable band gaps, while 2D materials provide smooth interfaces and channels with superior charge carrier mobility. However, the potential of devices utilizing QDs as floating gates and 2D materials as channels remains largely underexplored. Herein, a floating‐gate phototransistor based on the mixed‐dimensional heterostructure of 0D‐CsPbBr_3_ QDs and 2D‐MoS_2_ few layer is introduced. By leveraging the optical advantages of 0D‐QDs and the electrical properties of 2D materials, mixed‐modal in‐sensor reservoir computing (RC) is realized. Upon electrical/optical stimulation, the device demonstrates an on/off ratio of 10^7^, over 7‐bit multistates, nonlinear memory decay behavior, and dynamics with tunable time scales. Building upon these characteristics, the device enables mixed‐modal RC using mixed‐inputs of optical and electrical signals. Furthermore, accurate recognition of endangered species under extreme weather conditions is also demonstrated through audio‐visual fusion. This study presents a compelling paradigm for utilizing the properties of different‐dimensional materials to achieve mixed‐modal information fusion and opens new pathways for mimicking biological multisensory fusion.

## Graphical abstract

Designing bio‐inspired multisensory neuromorphic devices holds significant importance for low‐power and high‐efficiency computing. Here, the authors introduce a floating‐gate phototransistor based on the mixed‐dimensional heterostructure of 0D‐CsPbBr_3_ QDs and 2D‐MoS_2_ few layers. The device exhibits high‐accuracy endangered animal recognition under extreme environmental conditions through mixed‐modal reservoir computing (RC) and audio‐visual fusion mechanisms.

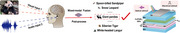

## 1 Introduction

The human brain exhibits efficient and low‐power parallel processing capabilities, enabling rapid responses to complex problems.^[^ ^1^, ^2^ ^]^ In dynamic environments, humans typically do not rely on a single sensory modality for information acquisition and decision‐making; rather, they depend on the complementarity and integration of multisensory inputs,^[^ ^3^, ^4^, ^5^ ^]^ particularly vision and hearing, which together account for over 90% of total perception.^[^ ^6^ ^]^ In this continuous learning process, synapses and neurons play a crucial role in the nervous system, promoting the optimization of cognition and perception.^[^ ^7^, ^8^ ^]^

In traditional sensor systems, perception, storage, and computation are mutually separate functions.^[^ ^9^, ^10^ ^]^ This separation leads to substantial redundant data transmission between units, resulting in high latency and power consumption. Most current sensing systems convert diverse modal signals into a single type of electrical signal for processing. While this unifies the processing pathway, it leads to a surge in data volume, further exacerbating the system's high latency and energy consumption problems.^[^ ^11^, ^12^ ^]^ Additionally, reliance on single‐modal information sources significantly reduces recognition accuracy and increases the likelihood of misjudgment. These limitations are particularly pronounced in complex environments, highlighting the importance of mixed‐modal information fusion.^[^ ^13^ ^]^ Inspired by human multisensory fusion, artificial synaptic devices that integrating sensing, storage, and computation have been designed to mimic the efficient perception and mixed‐modal fusion capabilities of biological systems. These artificial synaptic devices can simultaneously process optical and electrical signals, reducing energy consumption caused by data redundancy and transmission, and possess preprocessing functions that enable rapid response and precise decision‐making in dynamic environments.^[^ ^14^, ^15^ ^]^ Compared to traditional devices limited to single‐modal signal processing, the in‐sensor computing hardware can enhance integration and process mixed‐modal information more efficiently.^[^ ^16^, ^17^, ^18^, ^19^ ^]^ Furthermore, most existing systems require additional edge computing devices for information processing, limiting the application potential of neuromorphic hardware.^[^ ^9^, ^10^ ^]^ The implementation of in‐sensor computing devices can overcome this limitation, further enhancing their prospects for widespread use.

Two‐dimensional (2D) materials, owing to their rich material types and the absence of surface dangling bonds at interfaces, can be vertically stacked to fabricate devices with various structures, such as floating‐gate field‐effect transistors (FGFETs)^[^ ^20^, ^21^, ^22^, ^23^, ^24^, ^25^, ^26^ ^]^ and ferroelectric field‐effect transistors (FeFETs).^[^ ^27^, ^28^ ^]^ Meanwhile, 2D materials like graphene and molybdenum disulfide (MoS_2_), with their ultra‐high carrier mobility, enable rapid charge transport and exhibit exceptional electrical performance.^[^ ^29^ ^]^ However, most of the previous research on 2D FGFETs has focused on their electrically controlled non‐volatile memory characteristics, processing only single‐modal electrical signals, such as simulating auditory and tactile signals.^[^ ^30^ ^]^ On the other hand, 0D quantum dots (QDs) possess strong quantum confinement effects, making them excellent candidates for light absorption and photoelectric conversion.^[^ ^31^, ^32^, ^33^ ^]^ Meanwhile, the absorption spectrum of QDs can be tuned by adjusting their size, allowing for light sensing at specific wavelengths.^[^ ^34^, ^35^ ^]^ This makes them ideal for simulating visual signals. Additionally, QDs possess strong charge‐trapping capabilities, making them well‐suited for use as floating‐gate materials in the floating‐gate phototransistor.^[^ ^36^, ^37^, ^38^ ^]^ In the previous studies, floating‐gate optoelectronic transistors based on perovskite QDs and organic material systems have been reported, demonstrating the advantages of perovskite QDs as a photosensitive floating‐gate layer in terms of light absorption and charge trapping.^[^ ^36^, ^38^ ^]^ However, most reported floating‐gate transistors based on perovskite QDs have primarily focused on their capability for optical pulse programming. By combining the outstanding electrical properties of 2D materials with the superior optical characteristics of 0D QDs, it is possible to construct mixed‐dimensional heterojunction FGFETs that efficiently process mixed‐modal and multisensory signals simultaneously.^[^ ^39^, ^40^, ^41^, ^42^, ^43^, ^44^ ^]^ The 0D‐2D floating‐gate devices not only provide significant advantages in mixed‐modal fusion, but also facilitate efficient information storage and preprocessing, providing a new solution for sensing and processing systems.

In this work, we present a floating‐gate phototransistor (FGPT) based on a 0D‐2D mixed‐dimensional heterostructure (0D‐2D FGPT), where 2D‐MoS_2_ serves as the semiconducting channel, thin 2D‐BN functions as the tunnel dielectric, and 0D‐CsPbBr_3_ QDs serve as the floating‐gate layer, respectively. We report on the volatile/non‐volatile memory behavior of the device by adjusting the stimulus intensity of electrical and optical controls. Under intense optical/electrical programming, the device exhibits a high on/off ratio (≈10⁷), multilevel storage (≈7 bits), and long retention stability (> 3000 s). Under weaker light illumination, the device displays nonlinear relaxation behavior and dynamics with tunable time‐scales. Leveraging these rich nonlinear decay behaviors, we designed a reservoir computing (RC) system to perform a mixed‐modal handwritten digit‐recognition task (based on the Modified National Institute of Standards and Technology (MNIST) database). Considering the complementary characteristics of different‐modal information in mixed‐modal fusion, we further simulated the audio‐visual fusion, demonstrating in‐sensor recognition of endangered animals under extreme weather conditions with a recognition accuracy exceeding 84.6%. Our demonstration of this multifunctional 0D‐2D FGPT device will enrich the field of low‐dimensional optoelectronics and thus paves the way for intelligent in‐sensor computing systems that imitate biological multisensory fusion.

## 2 Results and Discussion

### 2.1 Fabrication and Characterization of the MoS_2_/h‐BN/CsPbBr_3_ QDs Heterostructure

**Figure 1:**
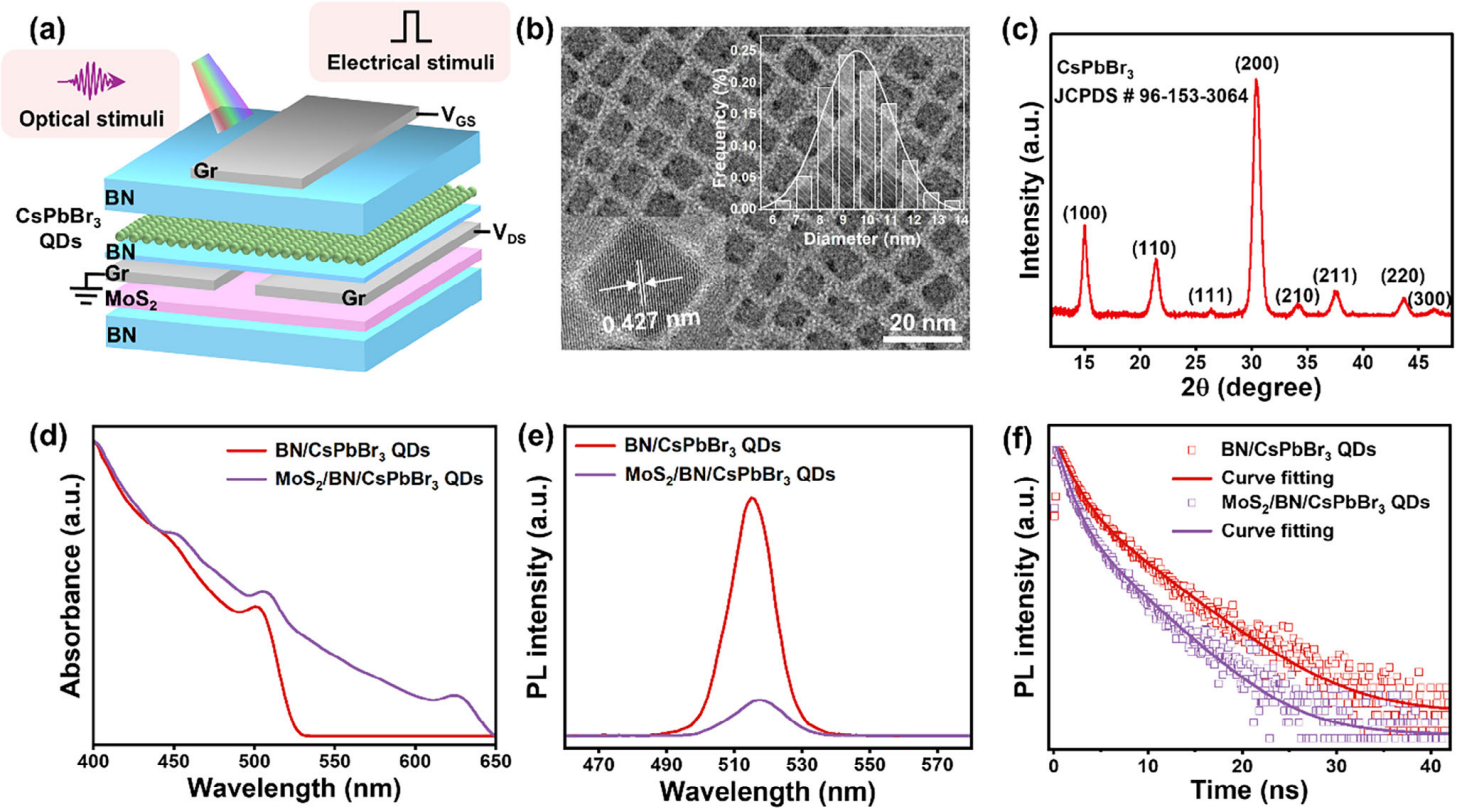
a) Schematic images of the 0D‐2D FGPT based on MoS_2_/h‐BN/CsPbBr_3_ QDs semiconductor heterostructure. b) TEM image of the CsPbBr_3_ QDs. Inset: HRTEM image and the size distribution of the QDs. c) XRD pattern of CsPbBr_3_ QDs. d) UV–visible absorption spectra of the MoS_2_/h‐BN/CsPbBr_3_ QDs and h‐BN/ CsPbBr_3_ QDs films. e) Steady‐state PL spectra of the MoS_2_/h‐BN/CsPbBr_3_ QDs and h‐BN/ CsPbBr_3_ QDs films on quartz (λ*ex*  =  402 *nm*). f) Transient PL spectra of the MoS_2_/h‐BN/CsPbBr_3_ QDs and h‐BN/ CsPbBr_3_ QDs films on quartz (λ*ex*  =  402 *nm*).

In this study, we first present a floating gate phototransistor based on the 0D‐2D hybrid‐dimensional heterostructure, which consists of 0D‐CsPbBr_3_ QDs as the FG, thin h‐BN as the tunnel dielectric, multilayer MoS_2_ as the semiconducting channel, and thick h‐BN /graphite as the gate dielectric/control gate, respectively, as illustrated in **Figure** **1**a. The source/drain electrodes were defined using thermal evaporation of Cr/Au (8/50 nm) on Graphene. The 0D‐2D FGPT is fabricated in the field‐effect architecture with the channel length and width of 3 and 5 µm. Details regarding the material preparation and heterostructure fabrication are provided in the Experimental Section and Figure S1 (Supporting Information).

The transmission electron microscope (TEM) image presents the monodispersed cubic shape CsPbBr_3_ QDs with an average diameter of ≈9.68 nm in Figure 1b. The statistical study of particle size dispersion fits a Gaussian distribution in the inset image of Figure 1b. Meanwhile, the high‐resolution transmission electron microscopy (HRTEM) image of the as‐prepared CsPbBr_3_ QDs illustrates clear lattice fringes with a spacing of 0.427 nm corresponding to the well‐arranged (200) planes of CsPbBr_3_ QDs. The X‐ray diffraction (XRD) pattern of CsPbBr_3_ QDs exhibited a typical cubic structure, corresponding to JCPDS#96‐153‐3064, indicating good crystallinity (Figure 1c). The diffraction peaks at 2*θ* = 15.0°, 21.4°, 26.3°, 30.3°, 34.2°, 37.7°, 43.6°, 46.4° correspond to (100), (110), (111), (200), (210), (211), (220), and (300) crystal planes of CsPbBr_3_ QDs, respectively.^[^ ^45^, ^46^ ^]^ Figure S2 (Supporting Information) shows that the spin‐coated CsPbBr_3_ QDs layer has a roughness of ≈5.60 nm and a thickness of around 20.45 nm. The atomic force microscope (AFM) was employed to measure the thicknesses of MoS_2_ (≈2.45 nm) and h‐BN (≈9.93 nm) film (Figure S3a,b, Supporting Information). Raman spectra were used to identify the involved films, as depicted in Figure S3c,d (Supporting Information). The h‐BN film was identified by the presence of the E_2 g_ lattice vibration mode at 1 363.14 cm^−1^. For MoS_2_, the Raman spectrum revealed characteristic E^1^_2 g_ and A_1g_ modes at 385.78 cm^−1^ and 404.79 cm^−1^, respectively.

The UV–vis absorption spectra of the h‐BN/CsPbBr_3_ QDs, and MoS_2_/h‐BN/CsPbBr_3_ QDs films are illustrated in Figure 1d. The h‐BN/CsPbBr_3_ QDs show an absorption cutoff around 532 nm, while MoS_2_/h‐BN/CsPbBr_3_ exhibits absorption in the red light region. Meanwhile, we measured the absorption spectrum of neat CsPbBr_3_ QDs, as shown in Figure S4 (Supporting Information). The steady‐state photoluminescence (PL) measurements can be employed to investigate the charge‐transfer efficiency. As shown in Figure 1e, upon excitation at 402 nm, the h‐BN/CsPbBr_3_ film exhibits a sharp emission peak centered at 515 nm. However, when MoS_2_ is combined with h‐BN/CsPbBr_3_, the PL intensity decreases significantly or is even quenched. This quenching originates from efficient charge transfer between MoS_2_ and h‐BN/CsPbBr_3_, indicating that highly effective exciton splitting has been achieved. Furthermore, the photoluminescence (PL) decay dynamics were fitted using a double‐exponential function to obtain the fluorescent lifetime parameters (Figure 1f). For the h‐BN/CsPbBr_3_ film, the fast component (τ_1_) and slow component (τ_2_) of PL lifetime parameters were found to be 1.45 and 5.45 ns. In contrast, the MoS_2_/h‐BN/CsPbBr_3_ film exhibited shorter PL lifetime parameters of τ₁  =  1.13 ns and τ₂  = 4.48 ns. The reduced PL lifetime parameters in the MoS_2_/h‐BN/CsPbBr_3_ film suggest that the presence of MoS_2_ accelerates exciton separation at the interface between MoS_2_/h‐BN/CsPbBr_3_, which is consistent with its decreased fluorescence intensity and indicating efficient charge transfer processes in the heterojunction film.^[^ ^47^ ^]^

### 2.2 Volatile/Nonvolatile Synaptic Behaviors of 0D‐2D FGPT Triggered by Optical Stimuli

**Figure 2:**
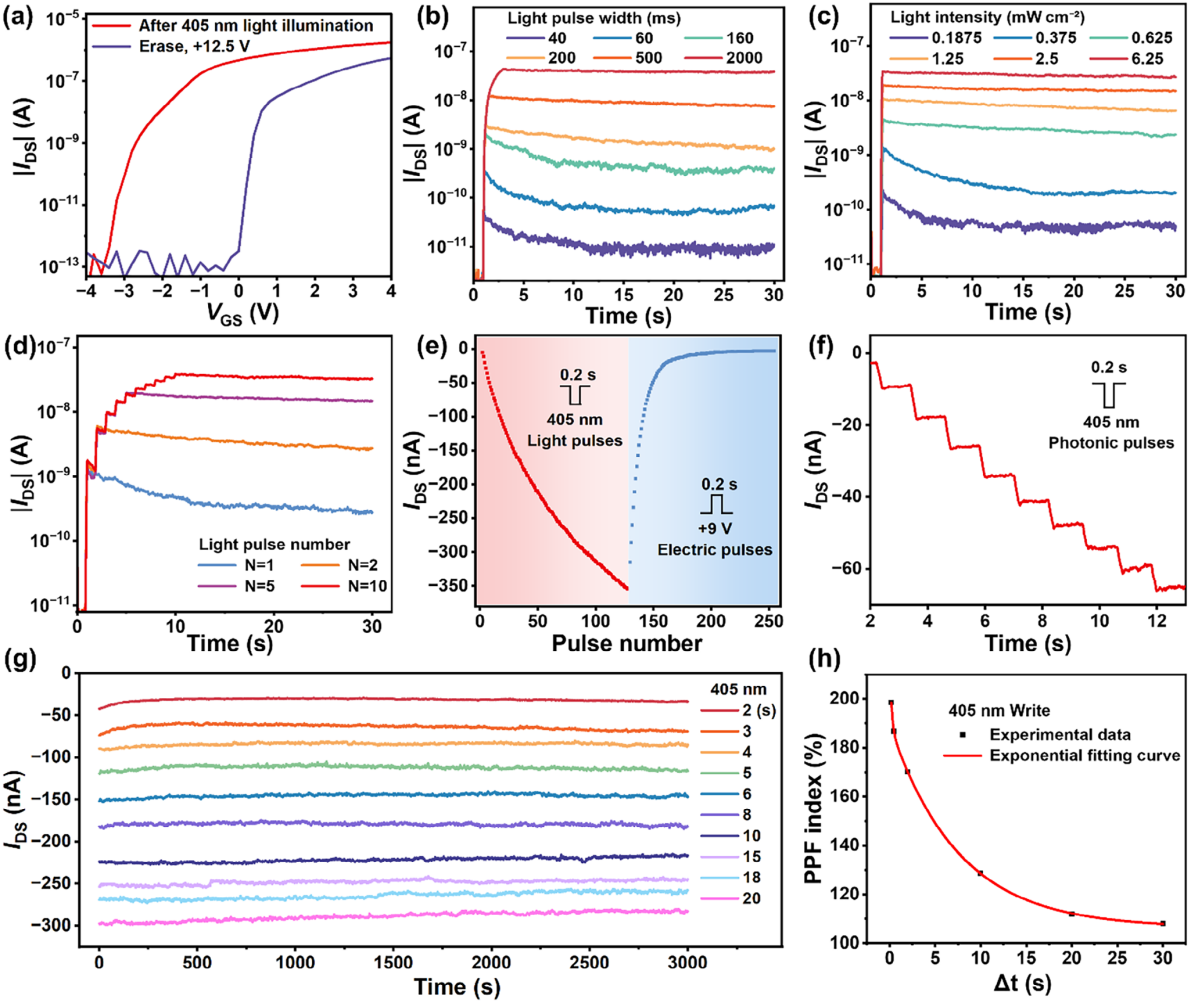
a) Transfer characteristics of the 0D‐2D FGPT in the light‐programing and electrical‐erasing states. b) The transition of STP to LTP triggered by increasing the width of the light pulse (405 nm, 0.375 mW cm^−2^). c)The transition of STP to LTP triggered by increasing the intensity of the light pulse ranging from 0.1875 to 6.25 mW cm^−2^ for 0.15 s. d) The transition of STP to LTP triggered by increasing the number of light pulses ranging from 1 to 10. e) Synaptic potentiation and depression behaviors induced through a series of light pulses (405 nm, 0.375 mW cm^−2^, 0.2 s) and positive electrical pulses (9 V, 0.2 s). f) Progressive I_DS_ modulation of the 0D‐2D FGPT induced through nine continuous light pulses (405 nm, 0.375 mW cm^−2^, 0.2 s). g) Retention stability of the 0D‐2D FGPT. The multi‐states were realized under different the width of the light pulse (405 nm, 0.375 mW cm^−2^) range from 2 s to 20 s. h) PPF behavior with different time intervals of paired light pulses ranging from 0.2 s to 30 s. The read bias is kept as –0.1 V in all the measurements.

To comprehensively evaluate the light pulse programing capability of the 0D‐2D FGPT, we characterized the transfer characteristics, synaptic behavior, and retention stability. **Figure** **2**a illustrates the transfer curve of the 0D‐2D FGPT after optical programing. Light pulse with a wavelength of 405 nm, an intensity of 0.375 mW cm^−2^, and a width of 2 s was employed as the programing operation, while the electrical pulse with a gate voltage of 12.5 V and a width of 1 s was used for the erasing operation. After the optical programing, the transfer curve shifts toward the negative direction, indicating that photogenerated holes are trapped in the CsPbBr_3_ QDs floating gate layer. Following the electrical erasing operation, the transfer curve shifts toward the positive direction, suggesting the release of the trapped holes from the floating gate layer. Additionally, we observed that the 0D‐2D FGPT achieves a memory window exceeding 3.5 V and an on/off current ratio of ≈10^7^. Meanwhile, Figure S5 (Supporting Information) shows the initial transfer curve of the 0D‐2D FGPT.

Figure S6 (Supporting Information) shows the variation of the drain‐source current (*I*_DS_) under a 405 nm light pulse. Upon stimulation by the light pulse, CsPbBr_3_ QDs absorb light and generate photogenerated carriers, causing *I*_DS_ to rapidly reach a maximum value. Subsequently, photogenerated holes trapped in shallow traps are prone to recombination, resulting in a nonlinear decay process. This dynamic behavior is analogous to the excitatory postsynaptic current (EPSC) observed in excitatory synapses of biological neural systems. Figure 2b–d and Figure S7 (Supporting Information) depict the EPSC responses of the 0D‐2D FGPT under different optical stimulation conditions. As shown in Figure 2b, the EPSC of the device increases as the width of the light pulse is extended. Specifically, longer light pulse durations lead to a greater EPSC response, indicating that the device's synaptic behavior is sensitive to the duration of optical stimulation. Similarly, increasing the light pulse power enhances the EPSC, demonstrating that the device's synaptic characteristics depend on the intensity of the optical stimulation (Figure 2c). Figure S8 (Supporting Information) shows the transfer characteristic curves after programing with light pulses of different intensities. As the light intensity increases, the threshold voltage gradually shifts toward more negative values. In addition, the EPSC shows a dependence on the frequency of the light pulses (Figure S7, Supporting Information). The EPSC is enhanced as the frequency increases, highlighting its potential for frequency‐based modulation of synaptic weight. Figure 2d illustrates that the EPSC of the device increases with the number of light pulses applied. As more light pulses are set, the EPSC shows a cumulative enhancement, suggesting that repeated optical stimulation can modulate the synaptic weight of 0D‐2D FGPT, similar to the synaptic plasticity observed in biological neural networks. Based on the above results, it is evident that when the light pulse parameters are relatively weak, the EPSC exhibits short‐term synaptic plasticity (STP). However, as the light pulse parameters are gradually intensified, the EPSC demonstrates long‐term synaptic plasticity (LTP).

Figure 2e depicts the optical programing and electrical erasing characteristics of the 0D‐2D FGPT, corresponding to the implementation of synaptic potentiation and depression, respectively. By setting a series of light pulses, the 0D‐2D FGPT achieves 128 distinguishable multistates, equiv. to 7‐bit information storage. This high degree of multilevel modulation demonstrates the capability for fine‐tuned synaptic weight adjustment. Furthermore, a selection of these multistates is shown in Figure 2f, indicating that the current level of the 0D‐2D FGPT has a strong linear correlation with the number of light pulses. This linear relationship demonstrates that the synaptic weight of the 0D‐2D FGPT can be precisely controlled through optical stimulation, which is crucial for applications in neuromorphic computing. On the other hand, Figure 2g demonstrates ten distinguishable current states with retention times exceeding 3 000 s, demonstrating the long‐term stability of the multistates memory in the 0D‐2D FGPT.

The EPSC of the 0D‐2D FGPT induced by a pair of light pulses with a time interval (Δt) of 0.5 s is depicted in Figure S9 (Supporting Information). The synaptic response to the second pulse results in a higher current compared to the first pulse, a phenomenon known as paired‐pulse facilitation (PPF) effect. The PPF index is calculated as:

(1)

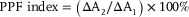

where ΔA₁ and ΔA₂ represent the differences in EPSC from the initial state after the first and second pulse, respectively.^[^ ^48^ ^]^ Figure 2h presents the PPF behavior with different time intervals of paired pulses ranging from 0.2 s to 30 s, indicating that the PPF index gradually decreases as the time interval increases. Fitting the results to a double‐exponential decay function:

(2) $$\mathrm{PPF}=C_{0}+C_{1}e^{-\Delta t/\tau_{1}}+C_{2}e^{-\Delta t/\tau_{2}}$$

as shown by the red line in Figure 2h, where τ_1_ = 0.20194 s and τ_2_ = 7.56639 s, respectively.^[^ ^49^ ^]^

To reveal the operation mechanisms of optical writing and electrical erasing, the energy band diagrams of the 0D‐2D FGPT under initial, optical‐programing, and electrical‐erasing conditions are illustrated in Figure S10 (Supporting Information).^[^ ^36^, ^37^, ^43^ ^]^ During the optical‐programing condition, a great number of photogenerated charge carriers are generated in the active layers of both the CsPbBr_3_ QDs and MoS_2_ layer. By bending the energy band, the photogenerated electrons in the CsPbBr_3_ QDs layer can easily tunnel through the h‐BN dielectric to the MoS_2_ channel, while the photogenerated holes remain confined in the valence band of the CsPbBr_3_ QDs. Upon removal of the light pulses, the photogenerated charge carriers in the MoS_2_ channel can recombine rapidly, causing an immediate decay of the EPSC in the initial phase. Meanwhile, the trapped holes in the CsPbBr_3_ QDs layer exhibit longer lifetimes, resulting in a slower decay of the subsequent EPSC behaviors.

### 2.3 Volatile/Nonvolatile Synaptic Behaviors of 0D‐2D FGPT Triggered by Electrical Stimuli

**Figure 3:**
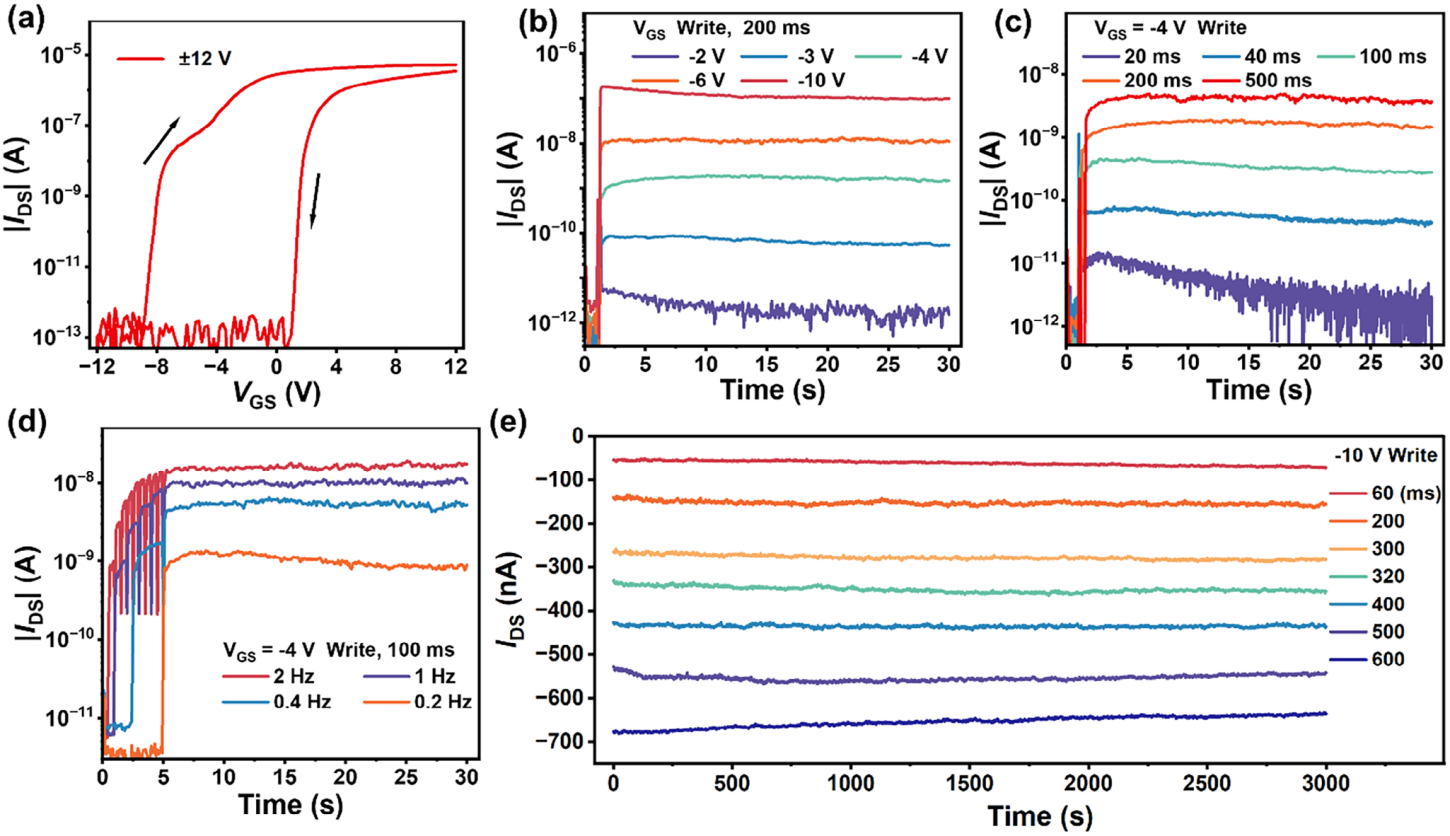
a) Transfer characteristics of the 0D‐2D FGPT in the electrical‐programing and electrical‐erasing states. I_DS_ evolution of the 0D‐2D FGPT tuned by negative electrical pulse trains with b) different V_GS_ at a fixed width of 200 ms, c) different width of the negative electrical pulse at a fixed V_GS_ of − 4 V, d) different frequencies of the negative electrical pulses at a fixed width of 100 ms and V_GS_ of −4 V. e) Retention stability of the 0D‐2D FGPT. The multi‐states were realized under different width of the electronic pulse (− 10 V) range from 0.06 to 0.6 s. The read bias is kept as − 0.1 V in all the measurements.

In addition to optical programing, the 0D‐2D FGPT can also perform write operations under negative gate voltage. **Figure** **3**a shows the transfer characteristics of the 0D‐2D FGPT after electrical programing. The device's threshold voltage undergoes significant shifts during both forward and reverse sweeps, thereby substantiating the charge storage capability of the CsPbBr_3_ QDs. Furthermore, to evaluate the device's writing capability under negative gate voltages, we extracted memory windows corresponding to different gate voltage scan ranges (5, 8, 10, and 12 V), as shown in Figure S11 (Supporting Information). Functional fitting of the test results revealed a strong linear correlation between the memory window and the range of gate voltage scans.

Figure 3b–d illustrates the EPSC responses of the 0D‐2D FGPT under different electrical stimulation conditions. As the intensity, pulse width, and frequency of electrical pulses increase, the 0D‐2D FGPT transitions from short‐term potentiation (STP) to long‐term potentiation (LTP). Additionally, we extracted the EPSC peak values for varying pulse widths and write gate voltages (Figures S12, Supporting Information). A linear relationship between the EPSC peak values and pulse width or write gate voltage was identified through functional fitting of the data.

Using the negative gate voltage programing and positive gate voltage erasing characteristics, the synaptic potentiation and depression behaviors were implemented in Figure S13 (Supporting Information). The train of negative gate voltage pulses (–4.5 V amplitude, 0.1 s duration, 1 s interval) applied onto the device increases the EPSC gradually. Consequently, positive gate voltage pulses (+ 10.1 V amplitude, 0.1 s duration, 1 s interval) are applied to mimic the electronic habituation in the 0D‐2D FGPT. By applying the set and reset operation of electric pulses, the device achieves multilevel synaptic weight tunability. Additionally, the long‐term storage capability was tested for seven distinguishable current states over 3 000 s in Figure 3e, confirming the long‐term storage stability of the 0D‐2D FGPT under electrical programing operations.

### 2.4 Mixed‐Modal In‐Sensor Computing Based on the 0D‐2D FGPT

**Figure 4:**
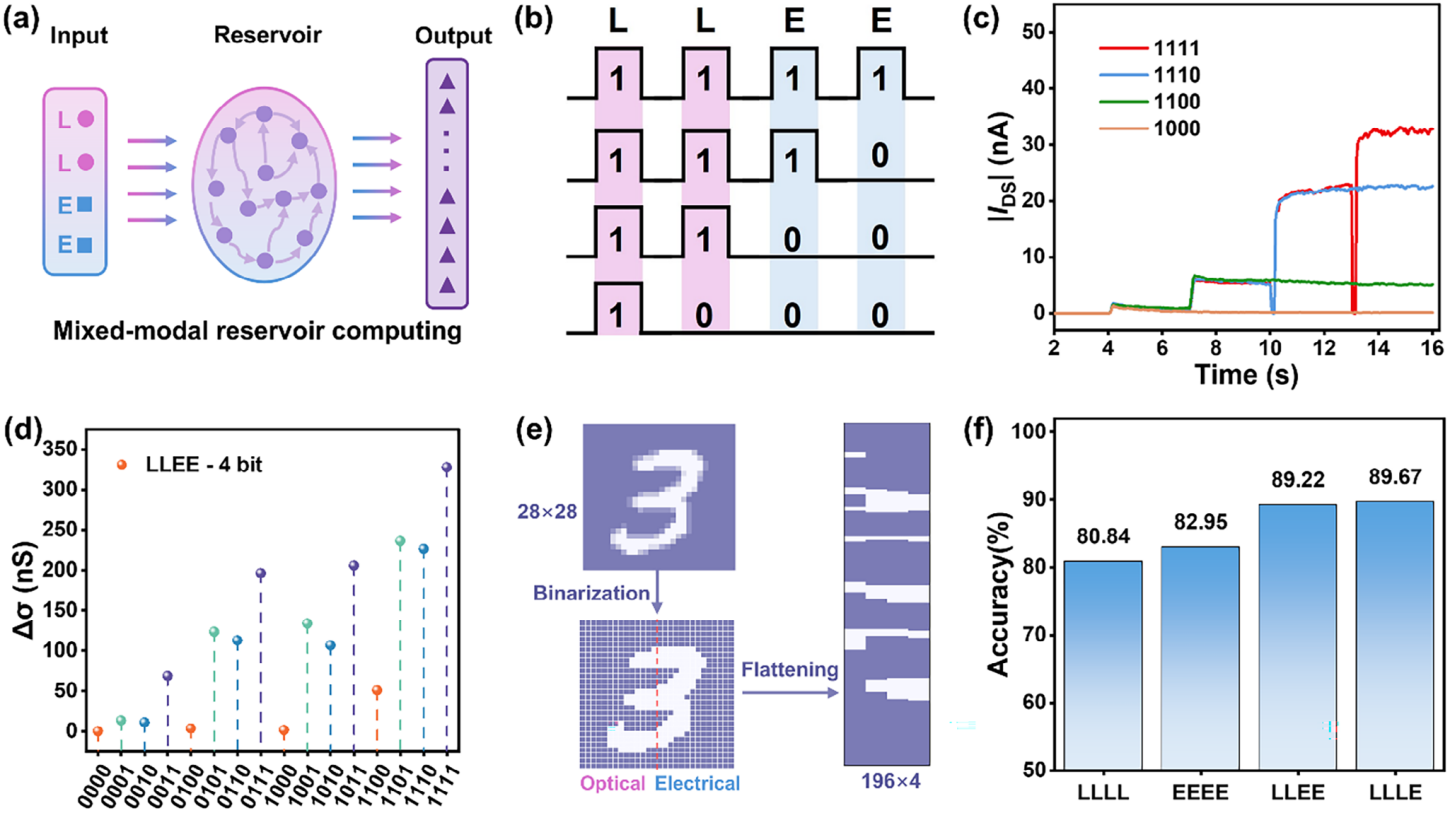
a) Schematics of different mixed‐modal fusion are carried out within a mixed‐input RC system. b) Demonstration of light and electrical pulse firing in 1111, 1110, 1100, 1000 combinations under the “LLEE” mode input. c) Response characteristics of the I_DS_ in 1111, 1110, 1100, 1000 combinations under the “LLEE” mode input (L: 405 nm, 0.375 mW cm^−2^, 0.15 s; E: − 3.75 V, 0.15 s). d) Channel conductance response generated by 16 different pulse sequences scoping from (0000) to (1111) under the “LLEE” mode input. e) Schematic illustration of the RC system employing optoelectronic hybrid inputs based on the MNIST database, incorporating processes such as binarization, optoelectronic encoding, and flattening. f) Recognition accuracy of the mixed‐mode digit‐recognition task at the “LLLL”, “EEEE”, “LLEE” and “LLLE” modes.

Considering that the 0D‐2D FGPT responds to both optical and electrical stimuli and exhibits rich nonlinear relaxation behavior and dynamics with tunable time‐scales, we have designed a RC system for mixed‐modal signal processing.^[^ ^50^, ^51^, ^52^ ^]^ Compared to traditional artificial neural networks (ANNs), RC offers advantages such as lower training costs and more efficient processing capabilities for temporal sequential data. In conventional mixed‐modal RC systems, each reservoir typically responds to only one type of input signal (e.g., LLLL/EEEE). Integration and classification of different modalities occur at the output layer. However, the 0D‐2D FGPT's ability to respond to both optical and electrical stimuli (e.g., LLLE/LLEE) means that feature extraction from different types of input signals can be performed within the same reservoir (**Figure** **4**a). This capability enhances signal processing efficiency and reduces energy consumption.

To evaluate the mixed‐modal in‐sensor computing capability of the optoelectronic reservoirs, we applied four different mixed input modes of 4‐bit binary streams: “LLLL”, “EEEE”, “LLEE”, and “LLLE”. As illustrated in Figure 4b, the “LLEE” mode indicates that the first two stimulus are light pulses (L), while the latter two are electrical pulses (E). Each square wave represents an input signal, where the “off” and “on” states of the light or electrical pulses correspond to “0” and “1”, respectively.^[^ ^13^, ^19^ ^]^ A light pulse representing “1” has an intensity of 0.375 mW cm⁻^2^ with a pulse width of 0.15 s, whereas an electrical pulse representing “1” applies a gate voltage of –3.75 V with a pulse width of 0.15 s. Figure 4c demonstrates the time‐dependent drain current variations for input sequences ranging from “0000” to “1111” under the “LLEE” mode. It can be observed that the final drain current state depends on the pulse type, sequence, and number. Figure 4d shows the conductance states encoded by 16 input combinations in the “LLEE” mode under the same initial conditions. Due to the reservoir's nonlinear relaxation and memory characteristics, the distributions of the 16 conductance states are distinguishable. The conductance states for the other three modes (“LLLL”, “EEEE”, and “LLLE”) are provided in Figure S14 (Supporting Information).

To demonstrate the mixed‐modal RC systems performance of the 0D‐2D FGPT, we conducted digit recognition tasks based on the MNIST dataset.^[^ ^17^, ^18^, ^26^ ^]^ In this proof‐of‐concept demonstration, conduction states measured from the device under diverse binary pulse sequences are integrated into a software‐based reservoir framework, effectively harnessing the optical and electrical responses of the 0D‐2D FGPT for classification. As shown in Figure 4e, using the handwritten digit “3” as an example, we illustrate the processes of image binarization, optoelectronic encoding, and flattening. The input image (28 × 28 pixels) is processed by grouping every four consecutive pixels in each column into a single unit vertically from top to bottom, resulting in an input matrix of dimensions 196 × 4. Each row of the matrix is then converted into pulses of different modes applied to the reservoir. For this task, we assume that the left half of the digit image can only be sensed by optical signals, while the right half can only be sensed by electrical signals. When the digit is input using a single‐mode reservoir (such as “LLLL” or “EEEE”), the system can only recognize part of the digit. Specifically, under the “LLLL” mode, only the left half of the image is recognized, while under the “EEEE” mode, only the right half is recognized. This limitation results in lower recognition accuracies when using only “LLLL” or “EEEE”, achieving 80.84% and 82.95%, respectively, as shown in Figure 4f. The mixed input reservoir modes (“LLEE” and “LLLE”) overcome this limitation by enabling recognition of the complete digit image. E.g., when the “LLEE” mode processes digits image, the “LL” portion responds to the left half of the digit, while the “EE” portion responds to the right half, achieving more comprehensive recognition. As shown in Figure 4f, higher recognition accuracies of 89.22% and 89.67% were obtained using the “LLEE” and “LLLE” modes, respectively. These results indicate that mixed input reservoir modes can effectively integrate information from different parts when processing complex mixed‐modal recognition tasks, significantly enhancing overall recognition performance.

### 2.5 Endangered Animal Monitoring System Based on Multisensory Fusion Reservoir Computing

Endangered animals are an indispensable part of ecosystems, and their conservation is of great significance for maintaining biodiversity and ecological balance. According to the 2024 data from the International Union for Conservation of Nature (IUCN), ≈45 300 species worldwide are currently listed as endangered or threatened, including over 26% of mammals and 41% of amphibians.^[^ ^53^, ^54^ ^]^ The rate of species extinction is accelerating at an unprecedented pace, exceeding the natural extinction rate by 100 to 1 000 times. From the perspective of the food chain and ecosystem, the extinction of a particular species can lead to the disruption of the food chain, affecting the survival of other species. E.g., the Siberian tiger, listed as an endangered species on the IUCN Red List, is a top predator that primarily preys on large herbivores such as sika deer, wild boar, and red deer. If the Siberian tiger were to become extinct, these herbivore populations would lose their natural predator, potentially leading to rapid population growth. This could result in overgrazing of forest vegetation by herbivores, leading to vegetation degradation. The reduction of vegetation not only directly impacts other animals inhabiting the forest, but also triggers broader ecosystem issues such as soil erosion and water resource depletion.^[^ ^55^, ^56^, ^57^ ^]^

To protect species diversity, various measures have been widely implemented, including artificial breeding, natural habitat preservation, and legislative protection.^[^ ^58^ ^]^ In the process of conserving endangered animals, monitoring technologies play a crucial role in understanding population trends and assessing degrees of endangerment. Among the most common methods are tagging and tracking,^[^ ^59^ ^]^ drones,^[^ ^60^ ^]^ remote sensing,^[^ ^61^ ^]^ camera traps,^[^ ^62^, ^63^ ^]^ and acoustic traps.^[^ ^64^ ^]^ Table S1 (Supporting Information) summarizes the advantages and disadvantages of these common monitoring methods.^[^ ^65^, ^66^ ^]^ Camera traps and acoustic traps enable long‐term monitoring of wildlife, while they generate redundant data and involve complex data analysis. At the same time, the trap method is easily disturbed by weather and environmental factors.

**Figure 5:**
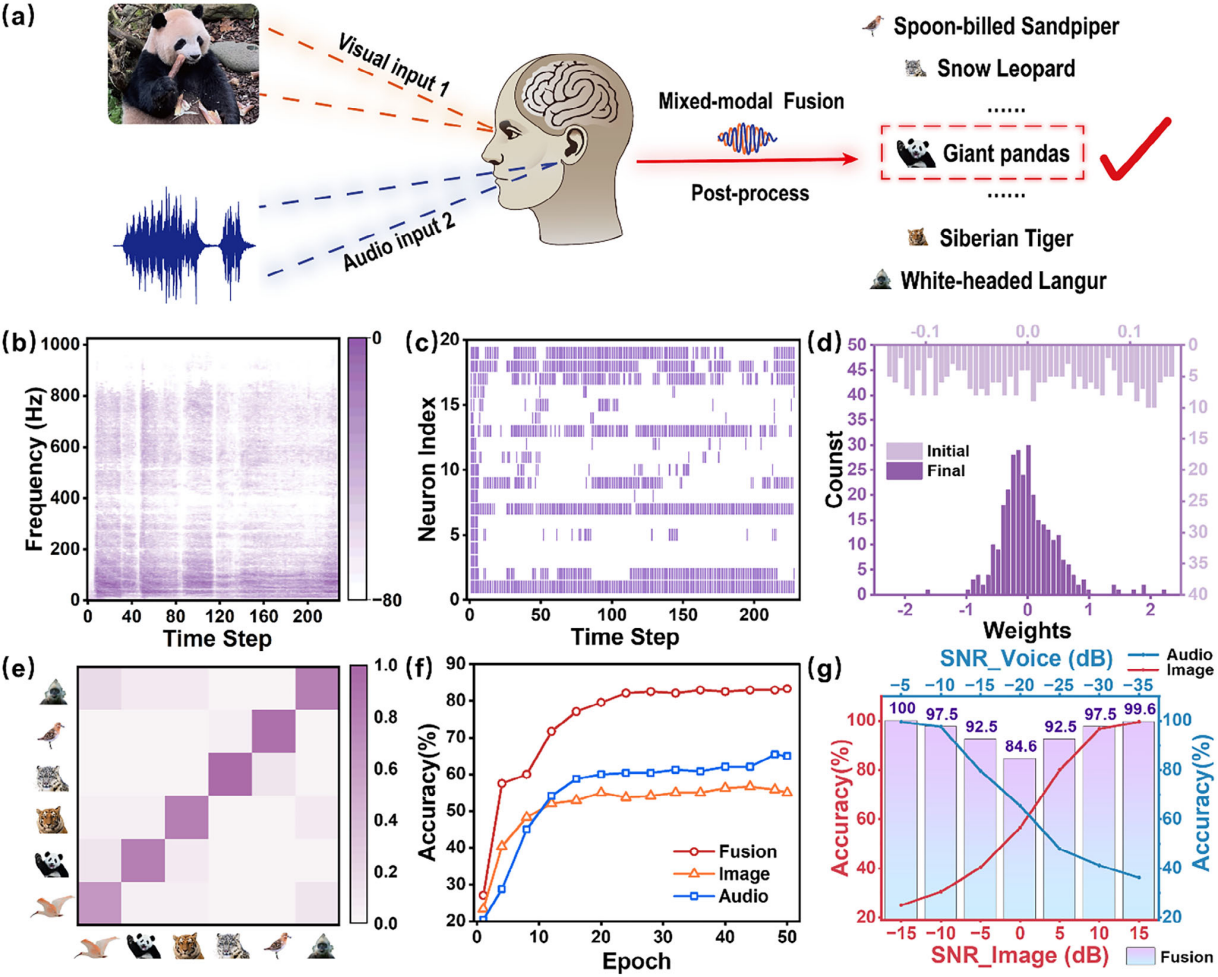
a) Schematics of using the 0D‐2D FGPT to achieve endangered animal recognition through audio‐visual fusion. b) Power spectrum of the audio, reflecting the frequency domain distribution of the audio over time. c) Sample of firing pulses of the time‐surface neurons from the first 20 channels in the encoding layer. d) Initial/final synaptic weight distributions before and after training when an audio SNR of − 20  dB and an image SNR of 0 dB. e) Confusion matrix on classifying the endangered animal test set in the mixed‐modal fusion mode. f) Comparison of recognition accuracy of the endangered animal recognition task under single‐modal and mixed‐modal information processing. g) Recognition accuracy of the endangered animal recognition task under different SNRs.

Inspired by human multisensory integration, we propose using the 0D‐2D FGPT to achieve endangered animal recognition through audio‐visual fusion (**Figure** **5**a). In this scheme, the audio and video datasets are processed by their respective reservoirs, and the resulting feature representations are subsequently integrated and analyzed by the neural network at the following layer. This method allows for high‐precision recognition tasks by using sound and visual signals to complement each other, especially in extreme weather conditions. Additionally, data analysis is directly implemented within the sensing hardware, thereby avoiding the issues of large data volumes and complex analyses associated with current camera and acoustic traps.^[^ ^67^ ^]^

To simulate environmental interference caused by weather conditions, we introduced noise into the data. Specifically, we added noise to the image data to mimic foggy weather and added noise to the audio data to simulate the sound of rainfall. To evaluate our system, we collected audio samples of six different wild animals from public sound sources, with each sample duration ranging from 1.02 to 5.29 s. Due to the presence of repetitive information in the audio data and to reduce computational complexity, we standardized the selection to a 1‐s segment of effective information from each sample. By introducing noise with different signal‐to‐noise ratios (SNRs) to augment the sample size, we increased the total number of samples to 1 200, of which 960 were used for the training set and 240 for the test set. Figure 5b displays the power spectrum of an audio segment, illustrating the frequency domain distribution of the audio over time. The power spectrum was converted into a Mel spectrogram using a Mel filter bank in Figure S15 (Supporting Information). At the same time, we extracted the Mel‐frequency cepstral coefficients (MFCCs).^[^ ^68^ ^]^ Since most of the effective data are concentrated in the low‐frequency region after applying the discrete cosine transform, we selected only the information from the first 20 channels. The data were then binarized and finally converted into event data, as shown in Figure 5c. Similarly, the image dataset was also obtained from public databases, and its processing method was the same as that used for the MNIST data processing described earlier.

Figure 5d–f presents the test data obtained at an audio SNR of –20 dB and an image SNR of 0 dB. The initial and final weight distributions under the mixed‐modal fusion mode are shown in Figure 5d. After training, the synaptic weights transitioned from a random distribution to a quasinormal distribution, indicating effective neural network training. Figure 5e displays the confusion matrix for classifying the test set in the mixed‐modal fusion mode. After 50 epochs, the recognition accuracies using only images or only audio were both below 65%, demonstrating that recognizing different endangered animal species using single‐modal information under high noise conditions is challenging (Figure 5f). In contrast, the recognition accuracy reached 84.6% using multisensory fusion. Clearly, these results indicate that the multisensory fusion method based on the 0D‐2D FGPT offers significant advantages in recognizing endangered animals under adverse weather conditions.

To further demonstrate the superiority of our multisensory fusion RC system, we tested the recognition accuracy under different SNRs for audio and images (Figure 5g). As noise levels increased, the recognition accuracy using only audio decreased from 99.58% to 36.25%, while using only images decreased from 99.58% to 25%. It is evident that under severe environmental interference, the recognition accuracy for endangered animals using only single‐modal information is very low, severely affecting species population statistics. However, in the multisensory fusion mode, by complementing audio and image information, the recognition accuracy remained above 84.6% in large environmental noise. Therefore, utilizing the multisensory fusion method based on the 0D‐2D FGPT not only avoids the issue of data redundancy at the sensing hardware level, but also enables accurate recognition of endangered animals under conditions of extreme environmental interference.

## 3 Conclusion

We report a floating‐gate phototransistor based on a hybrid‐dimensional heterojunction of 0D QDs and 2D materials. Benefiting from the high photoelectric conversion efficiency and excellent charge‐trapping capability of perovskite QDs, the device exhibits outstanding nonvolatile memory behavior under strong optical and electrical pulse stimulation, with features such as a high on/off ratio (≈10⁷), multiple storage states (≈7 bits), and long retention stability (> 3 000 s). Additionally, when the stimuli are weakened, the 0D‐2D FGPT displays rich tunable relaxation time and temporal dynamics. Based on these characteristics, we simulated a RC system to perform mixed‐modal handwritten digit recognition tasks on the MNIST dataset. Furthermore, leveraging the device's in‐sensor computing capabilities, we demonstrated a recognition accuracy exceeding 84.6% for endangered animal species under extreme weather interference using audio‐visual fusion, while also reducing data redundancy. This provides a novel technical approach for wildlife monitoring, opening new avenues for conserving species diversity and assessing species endangerment levels. More importantly, this work offers new design insights for future edge‐processing hardware systems with multisensory fusion.

## 4 Experimental Section

### Synthesis of CsPbBr_3_ QDs

The cesium oleate precursor was synthesized by loading 0.1628 g of Cs_2_CO_3_, 6 mL of octadecene (ODE), and 0.5 mL of oleic acid (OA) into a 50 mL three‐neck flask. The mixture was then heated under a nitrogen atmosphere to 160 °C to ensure complete reaction of Cs_2_CO_3_ with OA. The resulting solution was maintained at 160 °C to prevent further precipitation. In a separate three‐neck flask under vacuum conditions, 20 mL of ODE and 0.276 g of PbBr_2_ were loaded. The temperature was kept at 120 °C for 30 min and then raised to 180 °C under a nitrogen flow upon injection of 2 mL of oleylamine (OLAM) and 2 mL of OA. Once the temperature reached 180 °C, 1.6 mL of the cesium oleate precursor was immediately injected into the flask. After 5 s, the reaction was quenched by immersing the flask in an ice‐water bath, cooling the crude solution to room temperature. Subsequently, ethyl acetate was added to the crude solution at a ratio of 1:3 (crude solution: ethyl acetate), and the production was further centrifuged at 8 000 rpm for 10 min. The collected precipitate was then dispersed in n‐hexane, completing the first purification step. Ethyl acetate was added again to the dispersion at a ratio of 1:3 (n‐hexane dispersion:ethyl acetate), and the mixture was centrifuged at 8 000 rpm for 10 min to remove the supernatant. The obtained precipitate was then dispersed in n‐octane, completing the second purification step.

### Device Fabrication

MoS_2_, h‐BN, and Graphite nanoflakes were obtained from their maternal crystals through mechanical exfoliation. These nanoflakes were then transferred onto SiO_2_/Si substrates in a single step using polydimethylsiloxane (PDMS) and polycarbonate (PC) films. The electrodes were defined using ultraviolet photolithography, followed by thermal evaporation of Cr/Au (8 nm/50 nm). The synthesized CsPbBr_3_ QDs were spin‐coated at 3 000 rpm for 40 s and annealed at 100 °C for 40 min to remove the n‐hexane solvent. The h‐BN and Graphite were directly mechanically exfoliated onto PDMS and then sequentially transferred step‐by‐step onto the bottom mixed‐dimensional heterojunction using a dry transfer method.

### Characterization

The morphology and crystallization of as‐prepared CsPbBr_3_ QDs were characterized by TEM (Tecnai G2 F20 S‐TWIN, FEI). The crystallinity of CsPbBr_3_ QDs was characterized by XRD (Bruker D8 Advance). The UV–vis absorption spectra were recorded by the UV–vis Spectrophotometer (Energetiq EQ‐99X). PL spectra were obtained by Edinburgh Instruments (FLS 980) and a home‐built confocal microscope with excitation source from a tunable CW laser (400–2 300 nm, Photon etc). The height profiles of the vdW nanoflakes were characterized by AFM (Bruker, Dimension Icon with Scan Asyst), and the Raman spectra were obtained at room temperature using a LabRAM Odyssey Raman spectrometer with a polarized laser at a wavelength of 532 nm. All electrical characteristics of the 0D‐2D FGPT were measured by FS‐Pro 380 semiconductor device analyzer with devices placed in a vacuum probe station. Synergistic hybrid programing of electrical and light pulses was achieved by using one channel of the FS‐Pro 380 semiconductor device analyzer to drive a microcontroller controlling an optical shutter, along with a 405 nm monochromatic laser (Figure S16, Supporting Information).

## Conflict of Interest

The authors declare no conflict of interest.

## Supporting information



Supporting Information

## Data Availability

The data that support the findings of this study are available from the corresponding author upon reasonable request.
