# Supplementary material for: Mixed‐Dimensional Floating Gate Phototransistors for Mixed‐Modal In‐Sensor Reservoir Computing
Source: Adv Sci (Weinh). 2025 May 8;12(29):2502694. doi: 10.1002/advs.202502694 (PMC12362771; doi:10.1002/advs.202502694)
Supplement: Supplementary file 1 — Supporting Information [file ADVS-12-2502694-s001.docx]

Supporting Information

**Mixed-Dimensional Floating Gate Phototransistors for Mixed-Modal In-Sensor Reservoir Computing**

Weilun Ouyang, Qirui Zhang, Jiangang Chen, Xiao Luo*, Xuemei Wang, Yingying Chen, Fan Yang, Qi Nie, Qing Liu* and Fucai Liu*

**S1. Device fabrication process following the preparation sequence from bottom to top**

**S2 The thickness and uniformity of the perovskite CsPbBr_3_ QDs layer**

**S3. AFM and Raman characterization of MoS_2_ and h-BN**

**S4. The absorption spectrum curve of neat CsPbBr_3_ QDs**

**S5. Transfer curve of the 0D-2D FGPT in its initial state**

**S6. The variation of the drain-source current (*I*_ds_) under a 405 nm light pulse**

**S7. The EPSC shows a dependence on the frequency of the light pulses**

**S8. The transfer curves after programming with different light intensities**

**S9. The EPSC of the 0D-2D FGPT induced by a pair of light pulses with a time interval (Δt) of 0.5 s**

**S10. The operation mechanisms of optical writing and electrical erasing**

**S11. Memory windows corresponding to different gate voltage scan ranges**

**S12. The EPSC peak values for varying pulse widths and write gate voltages**

**S13. The synaptic potentiation and depression behaviors by negative gate voltage programming and positive gate voltage**

**S14. The conductance states for the other three modes ("LLLL", "EEEE", and "LLLE")**

**S15. A Mel spectrogram obtained from the power spectrum using a Mel filter bank**

**S16. Schematics of synergistic hybrid programming using electrical and light pulses**

**Table S1. The advantages and disadvantages of the common monitoring methods**


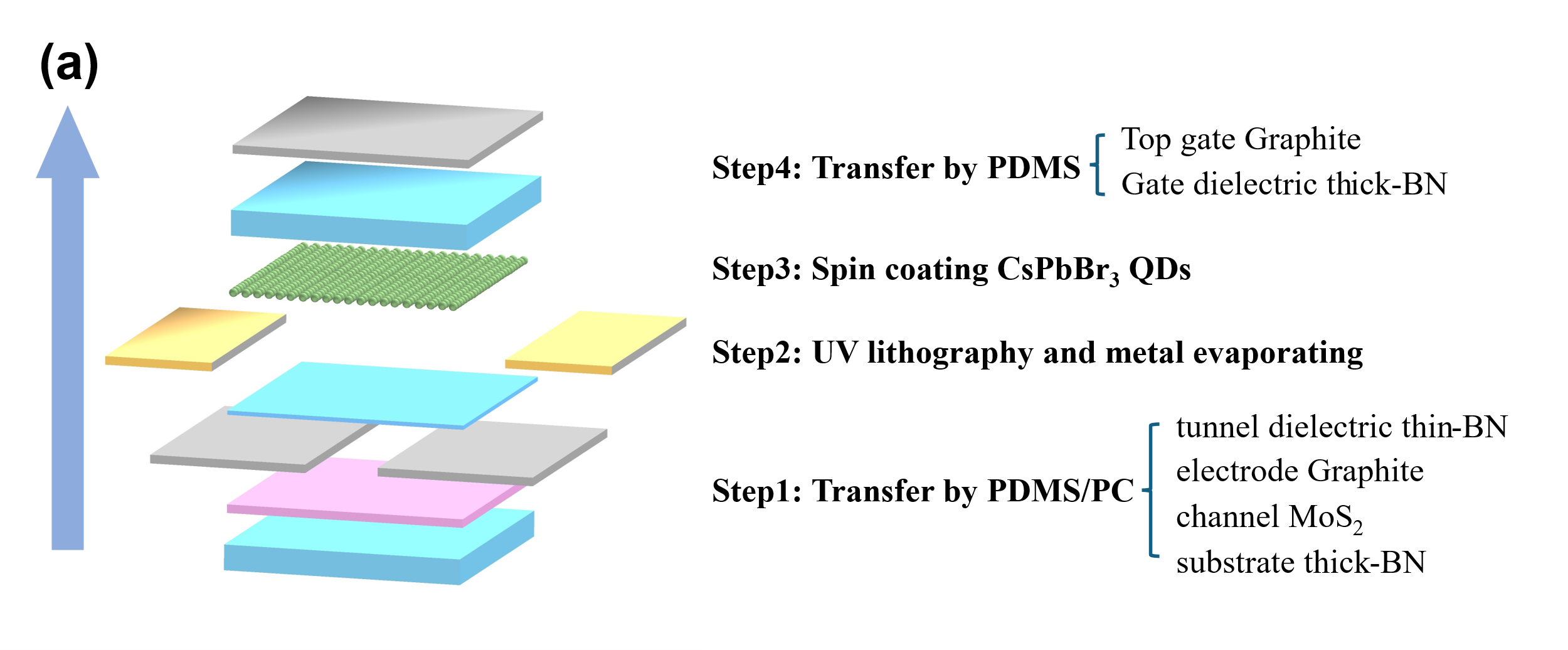


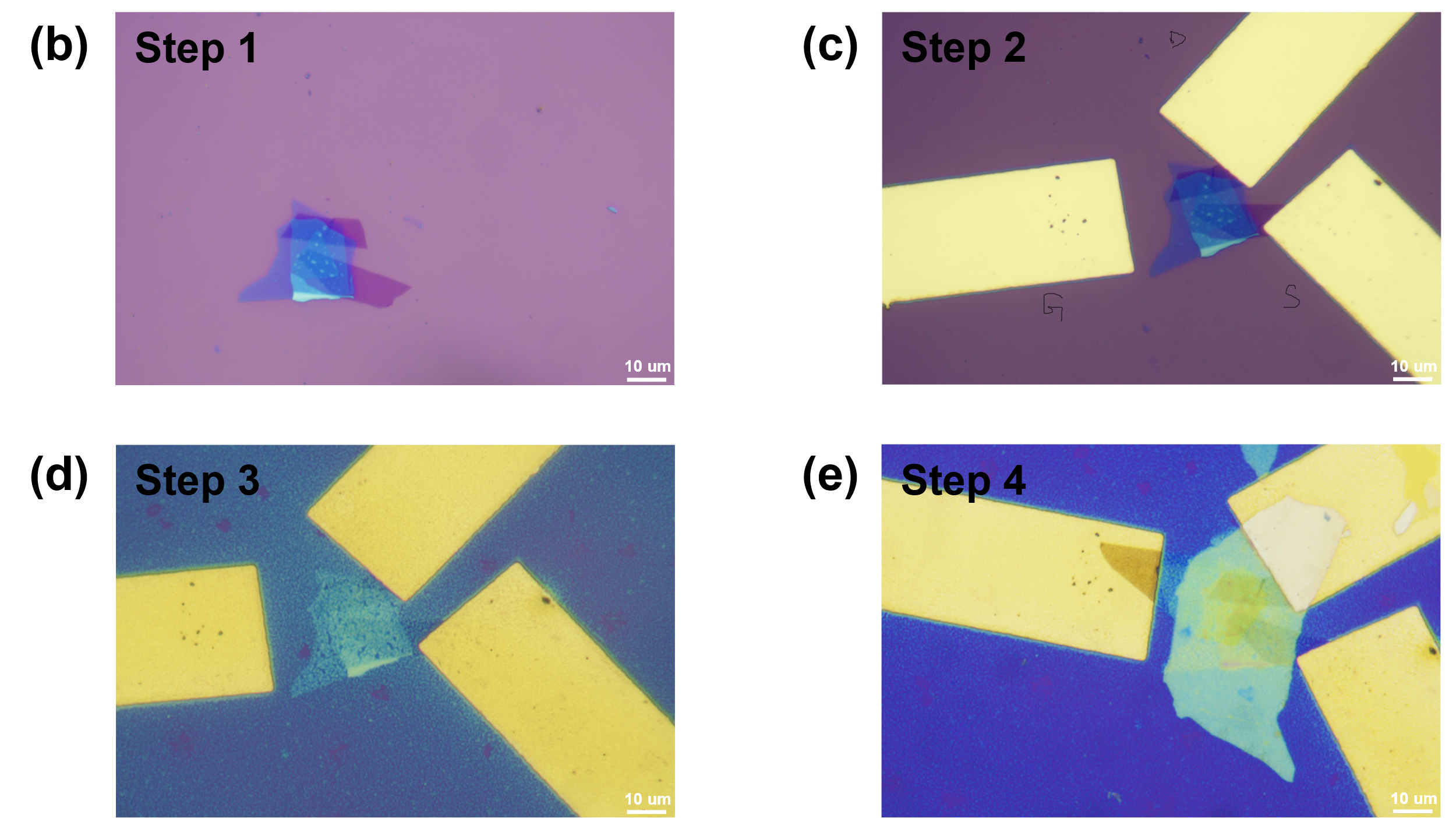


**Supplementary Figure 1.** Fabrication process of the 0D-2D FGPT. (a) Device fabrication process following the preparation sequence from bottom to top. (b) Step 1: Transferring the thick-BN/MoS_2_/Graphite/thin-BN on the SiO_2_/Si substrate by PDMS/PC. (c) Step 2: UV lithography and metal evaporating. (d) Step 3: Spin coating CsPbBr_3_ QDs. (e) Step 4: Transferring the gate dielectric thick-BN/top gate Graphite on the top of CsPbBr_3_ QDs by PDMS.


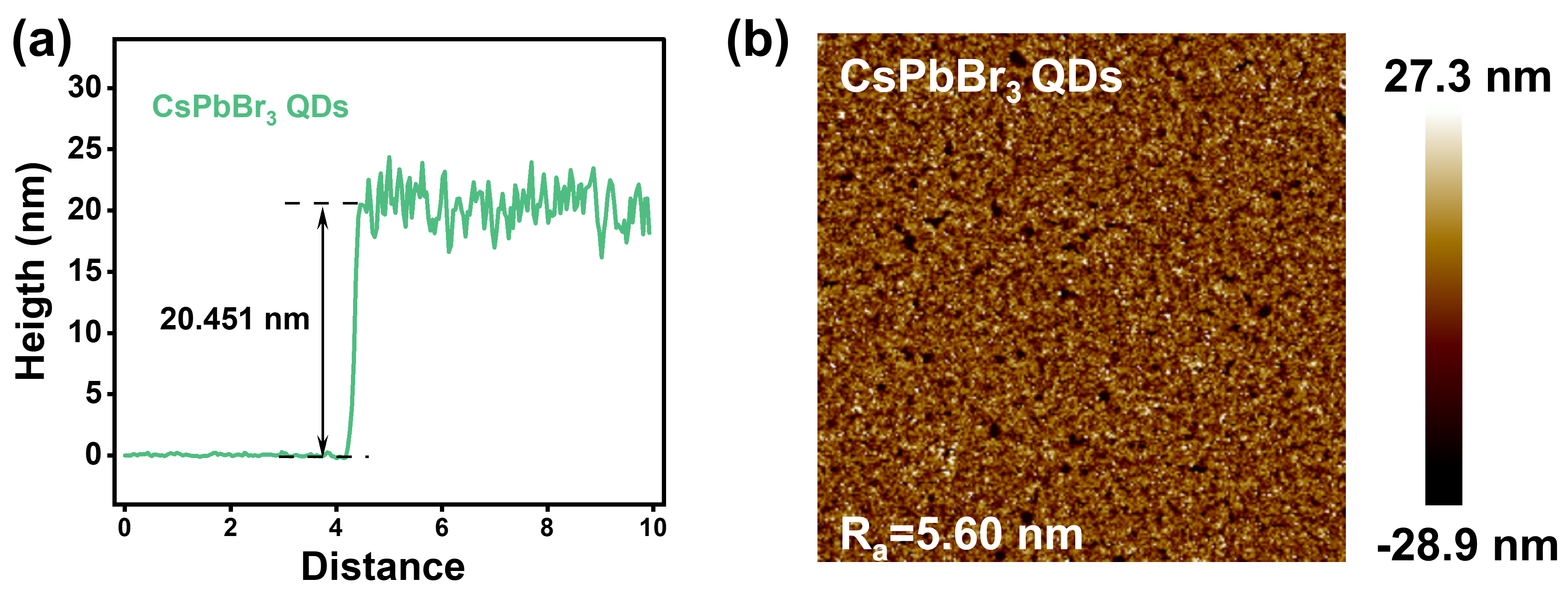


**Supplementary Figure 2.** The (a) thickness and (b) uniformity of the perovskite CsPbBr_3_ QDs layer.


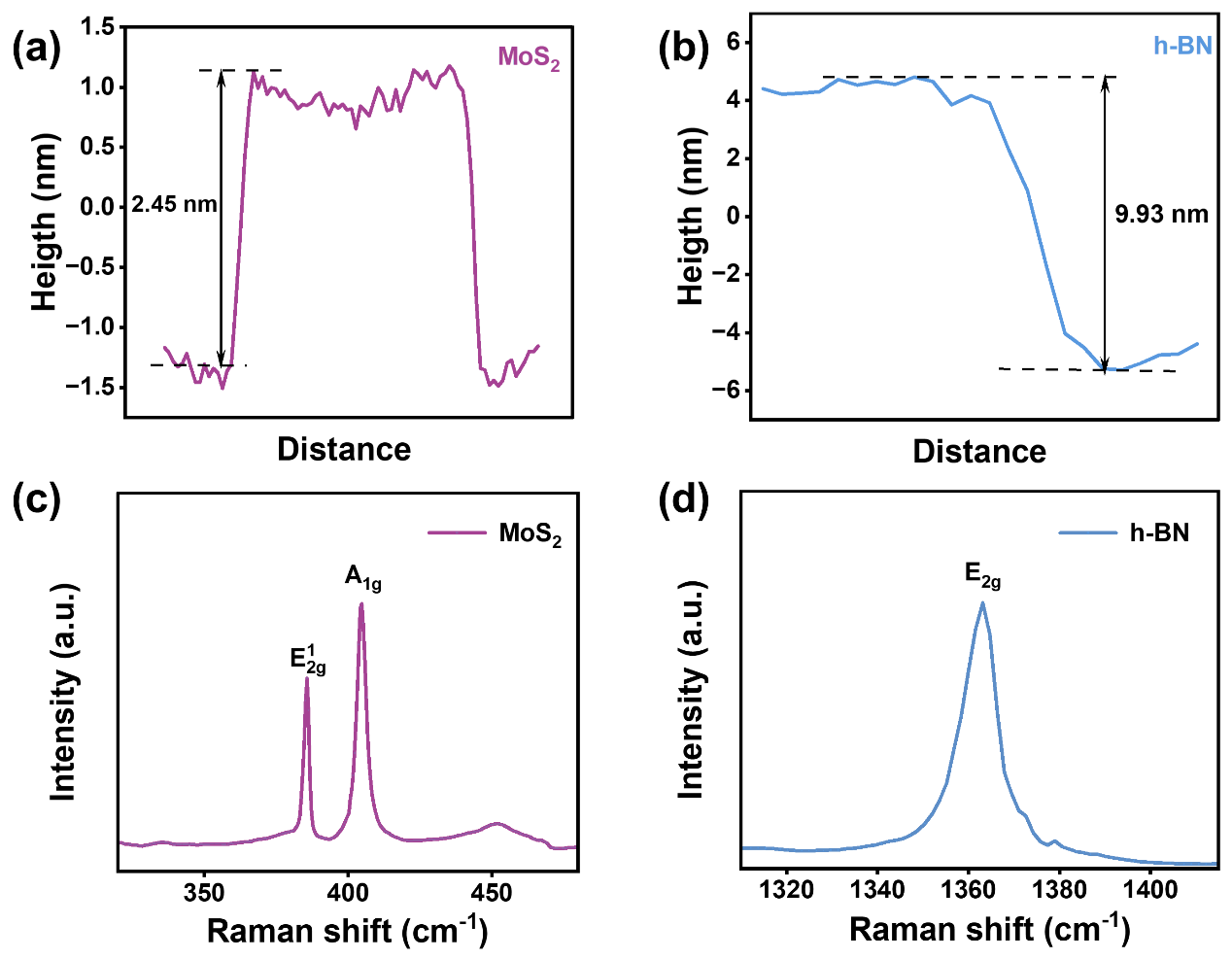


**Supplementary Figure 3.** AFM and Raman characterization of MoS_2_ and h-BN. (a) Height profile of the 2D MoS_2_ nanoflake, showing a thickness of ~2.45 nm. (b) Height profile of the 2D thin-BN nanoflake, showing a thickness of ~9.93 nm. (c) Raman spectra of the 2D MoS_2_ nanoflake. (d) Raman spectra of the 2D thin-BN nanoflake.


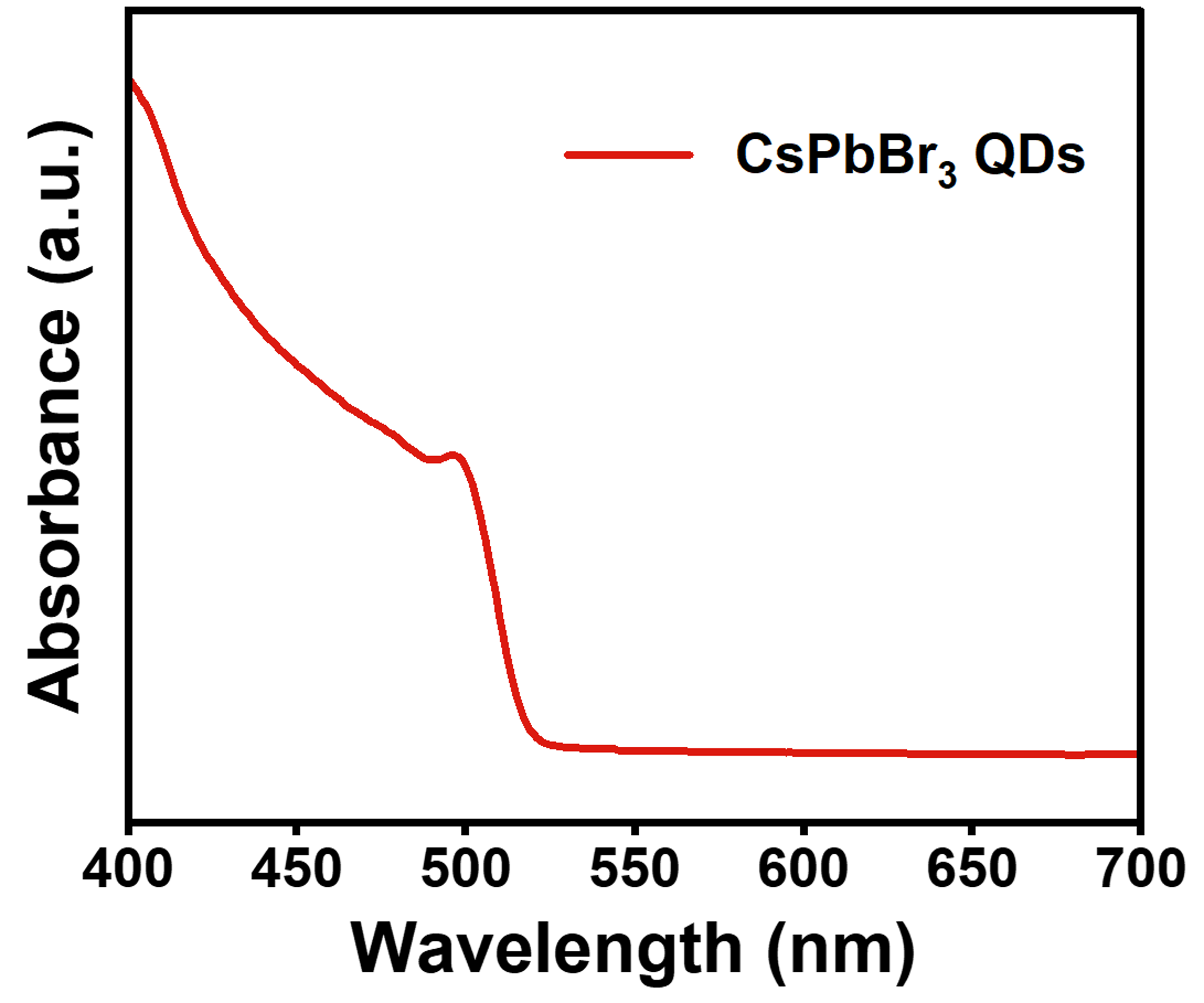


**Supplementary Figure 4.** The absorption spectrum curve of neat CsPbBr_3_ QDs.


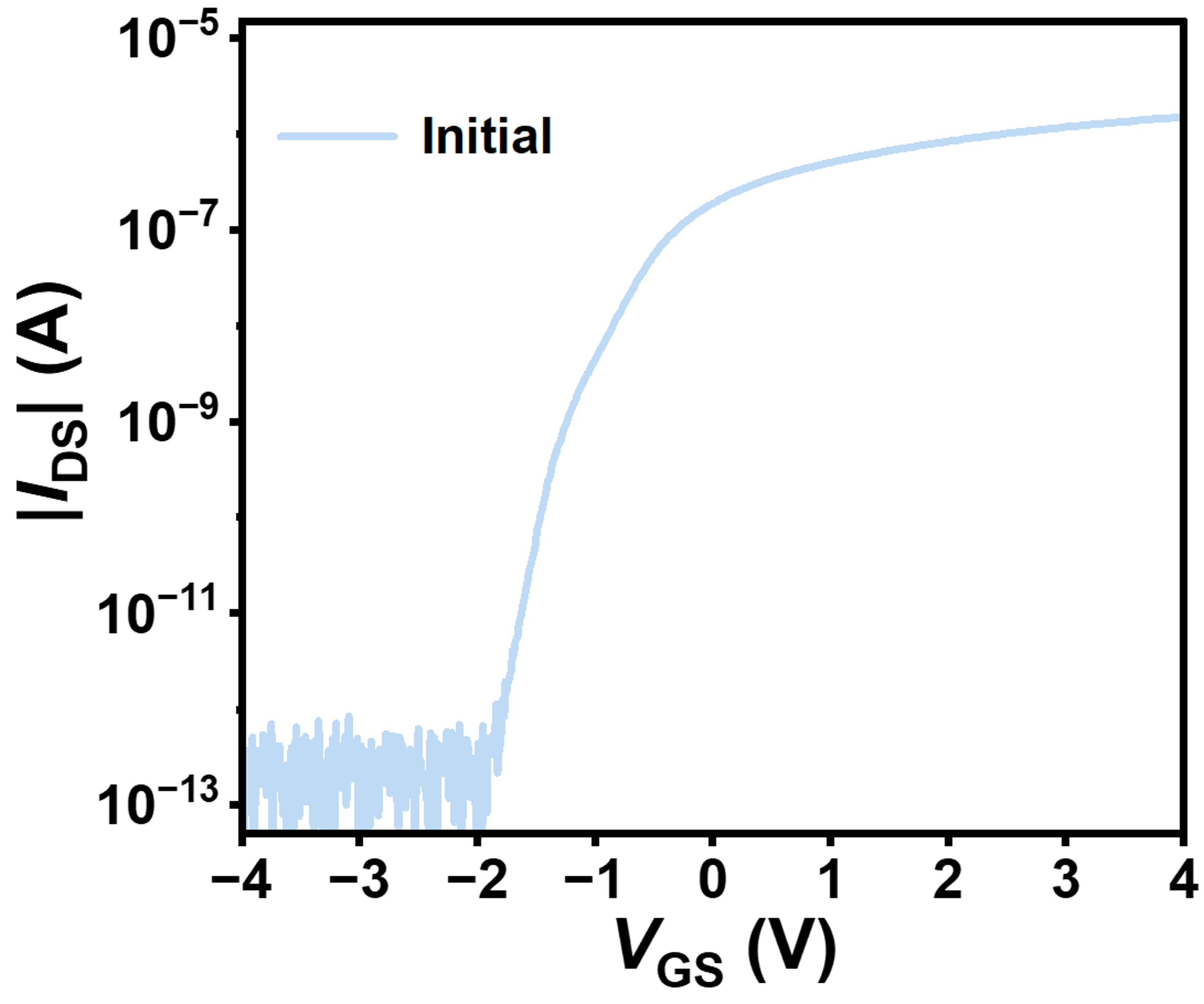


**Supplementary Figure 5.** Transfer curve of the 0D-2D FGPT in its initial state.


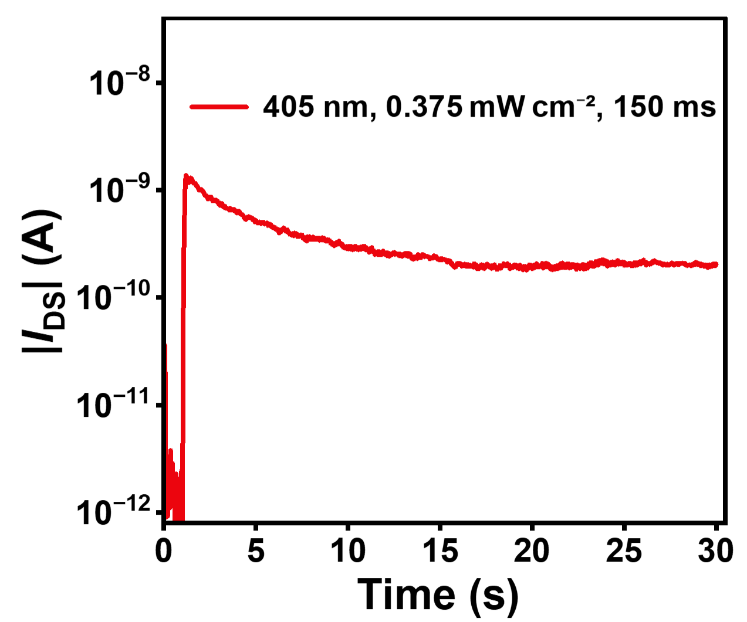


**Supplementary Figure 6.** The variation of the drain-source current (I_DS_) under a 405 nm light pulse with an intensity of 0.375 mW cm⁻², and a width of 150 ms.


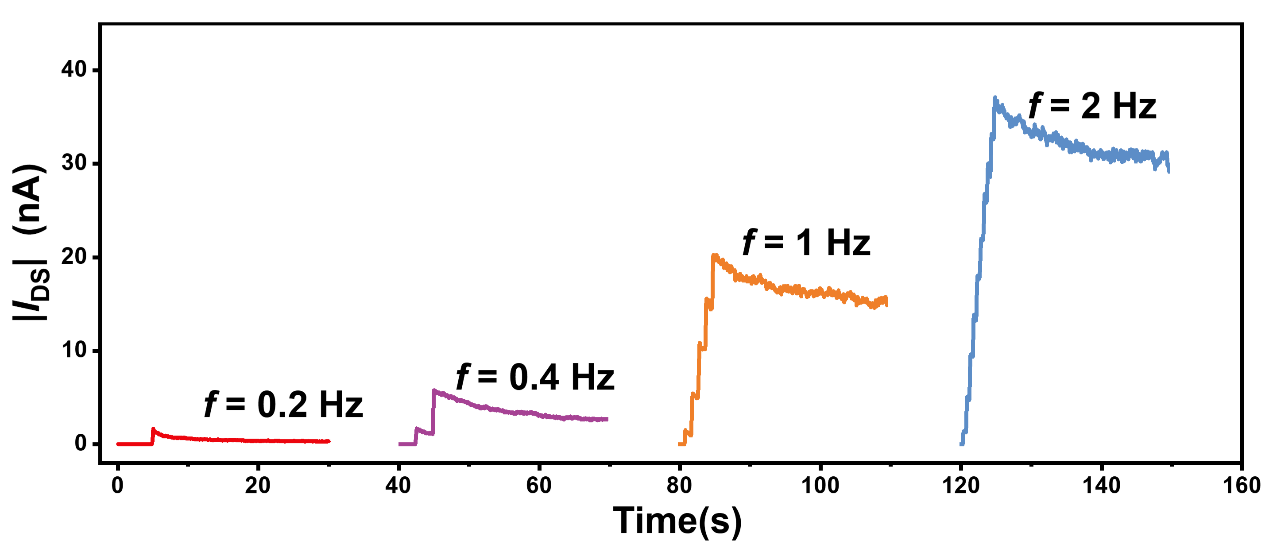


**Supplementary Figure 7.** The EPSC shows a frequency-dependent behavior when the light pulses are delivered at 0.2 Hz, 0.4 Hz, 1 Hz, and 2 Hz. The applied light pulses with a wavelength of 405 nm, an intensity of 0.375 mW cm^-2^, and a width of 150 ms.


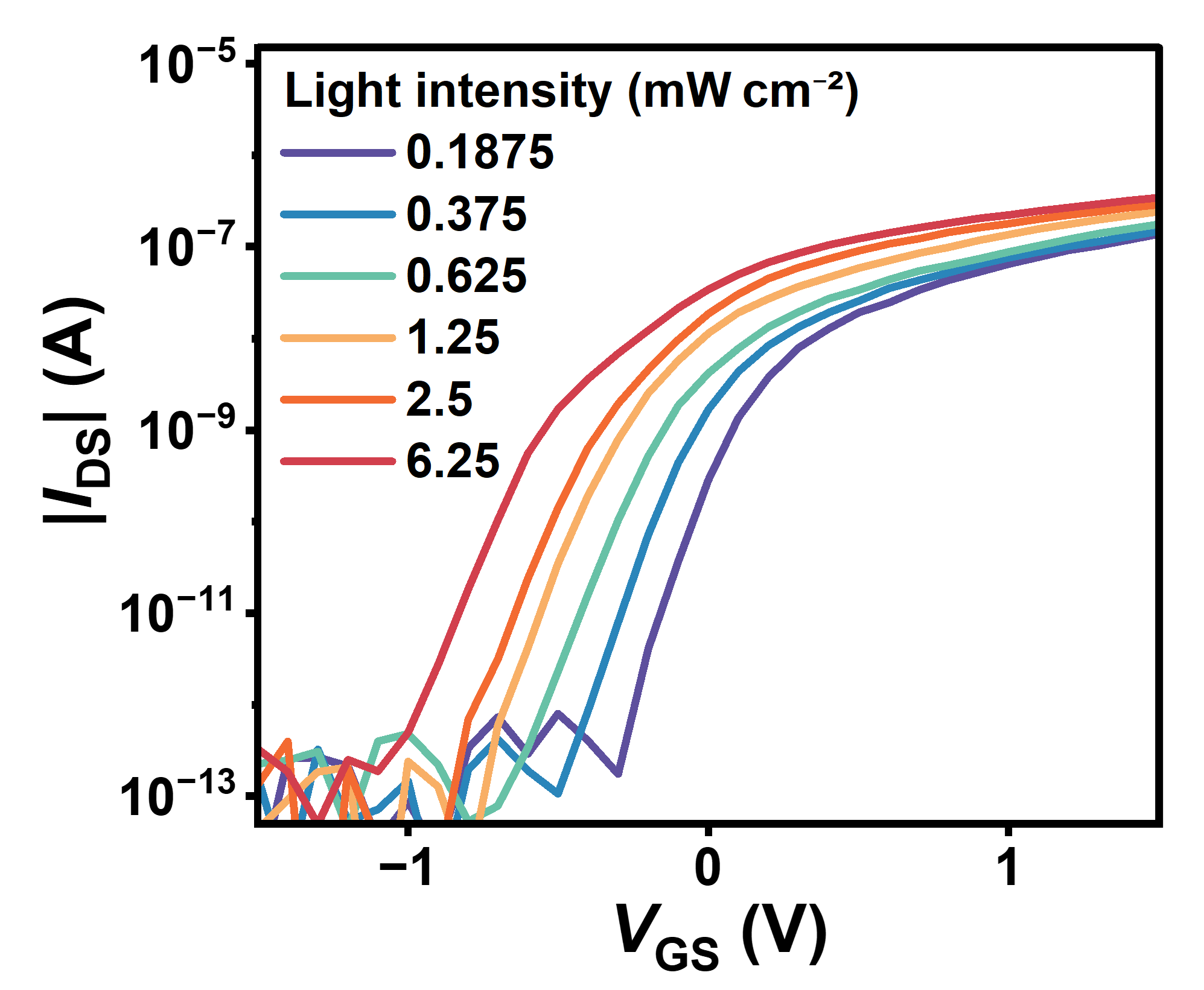


**Supplementary Figure 8.** Changes in the transfer characteristic curves after programming with light pulses at different intensities (ranging from 0.1875 to 6.25 mW cm^-2^ for 150 ms).


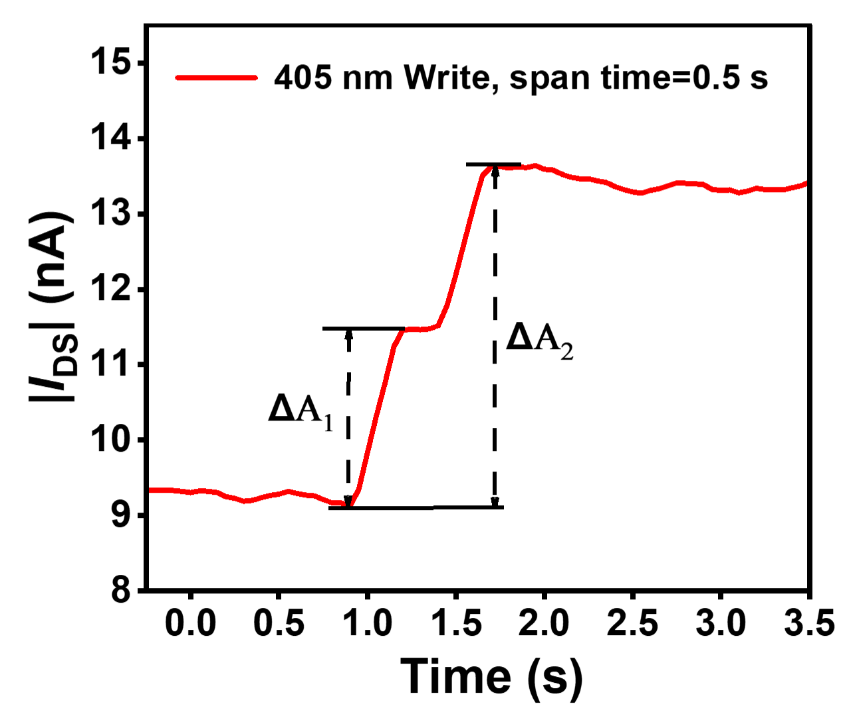


**Supplementary Figure 9.** The EPSC of the 0D-2D FGPT induced by a pair of light pulses with a time interval (Δt) of 0.5 s.


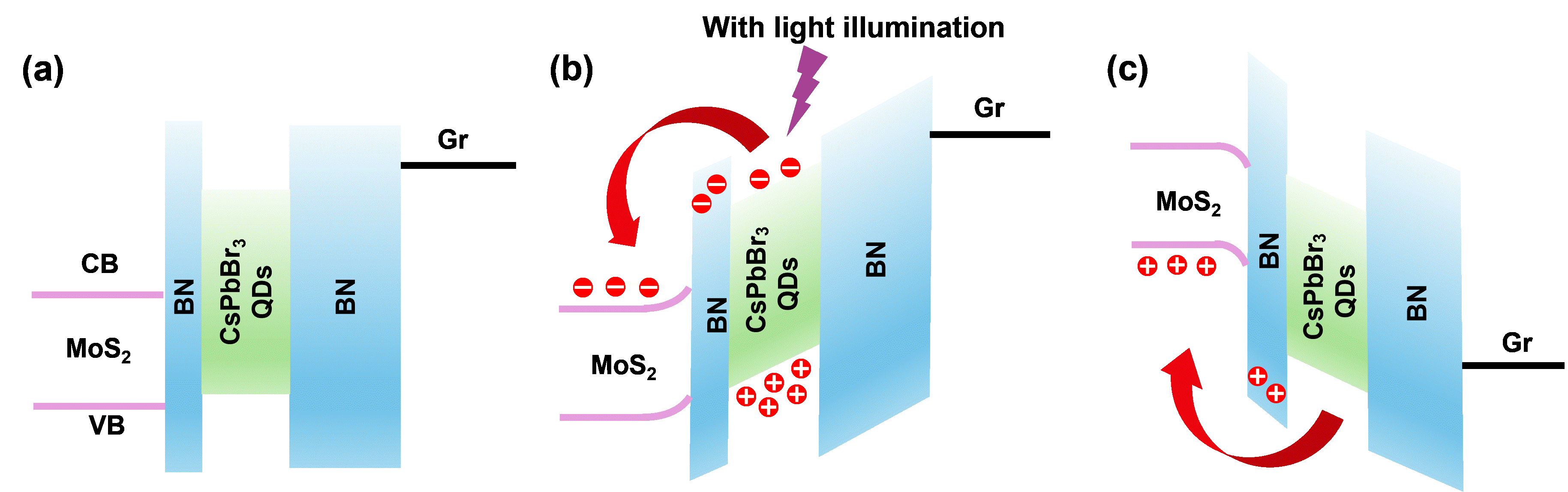


**Supplementary Figure 10.** The operation mechanisms of optical writing and electrical erasing, the energy band diagrams of the 0D-2D FGPT under (a) initial, (b) optical‐programming, and (c) electrical‐erasing conditions, respectively.


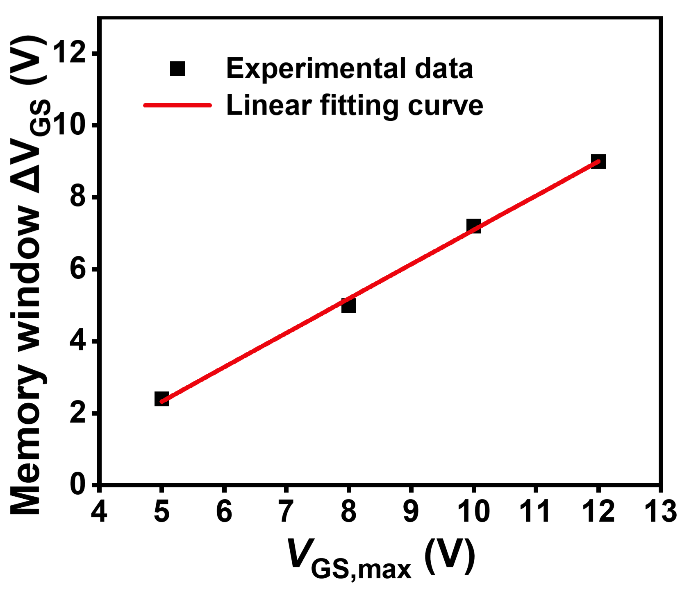


**Supplementary Figure 11.** The electrical memory windows correspond to different gate voltage scan ranges (5 V, 8 V, 10 V, and 12 V), and curve fitting revealed a strong linear correlation.


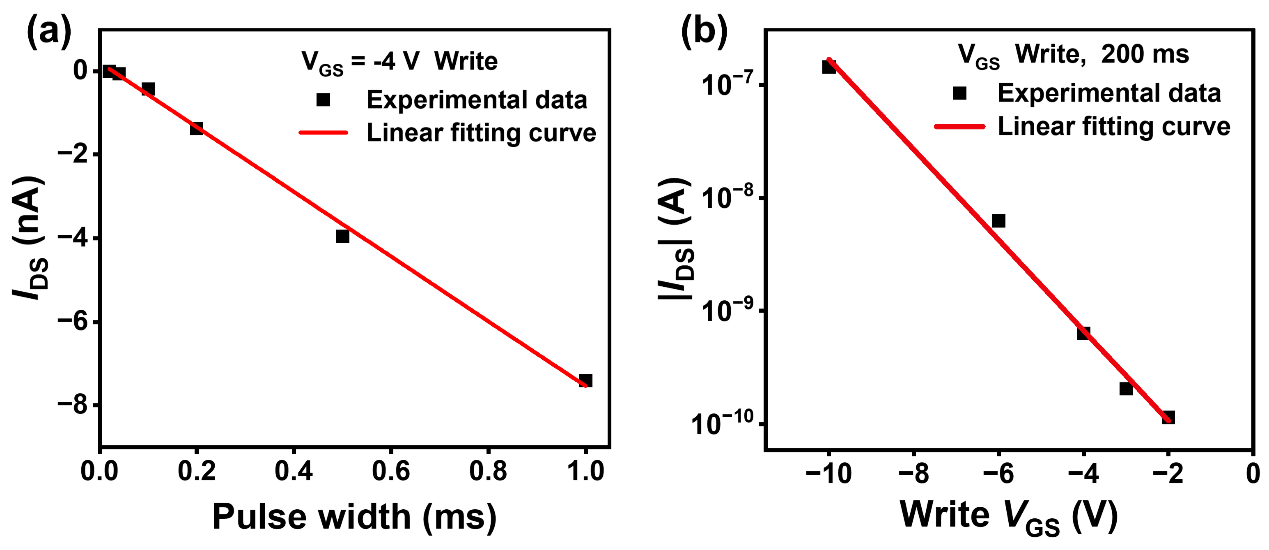


**Supplementary Figure 12.** The EPSC peak values for varying pulse under different conditions. (a) varying pulse widths at V_GS_ = -4V and (b) varying write gate voltages at a fixed pulse width of 200 ms. Additionally, fitting the data revealed a linear relationship between the EPSC peak values and both the pulse width and the write gate voltage.


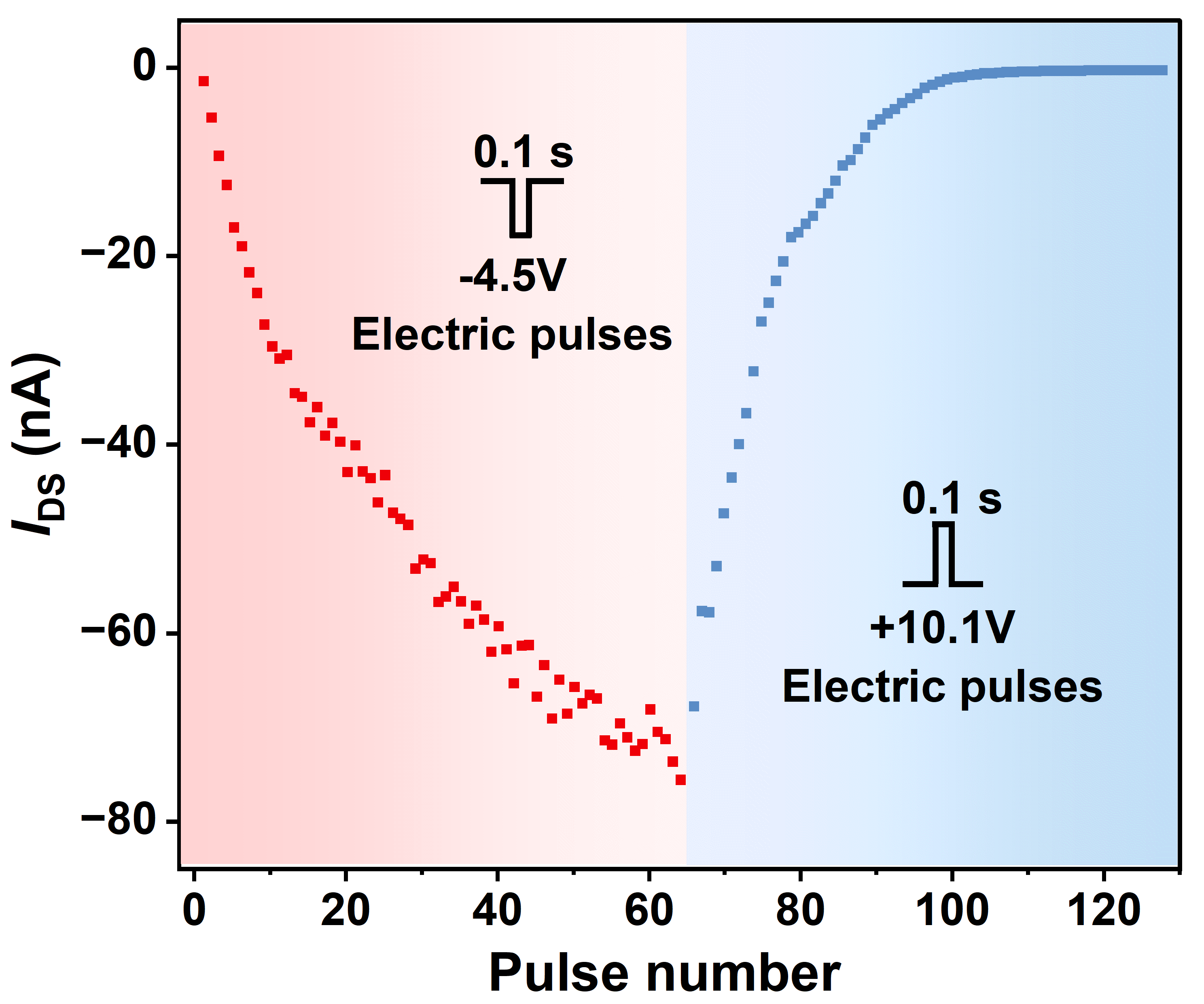


**Supplementary Figure 13.** The synaptic potentiation and depression behaviors induced by applying negative gate voltage pulses (–4 V, 100 ms) for programming and positive gate voltage pulses (+10.1 V, 100 ms) for erasing.


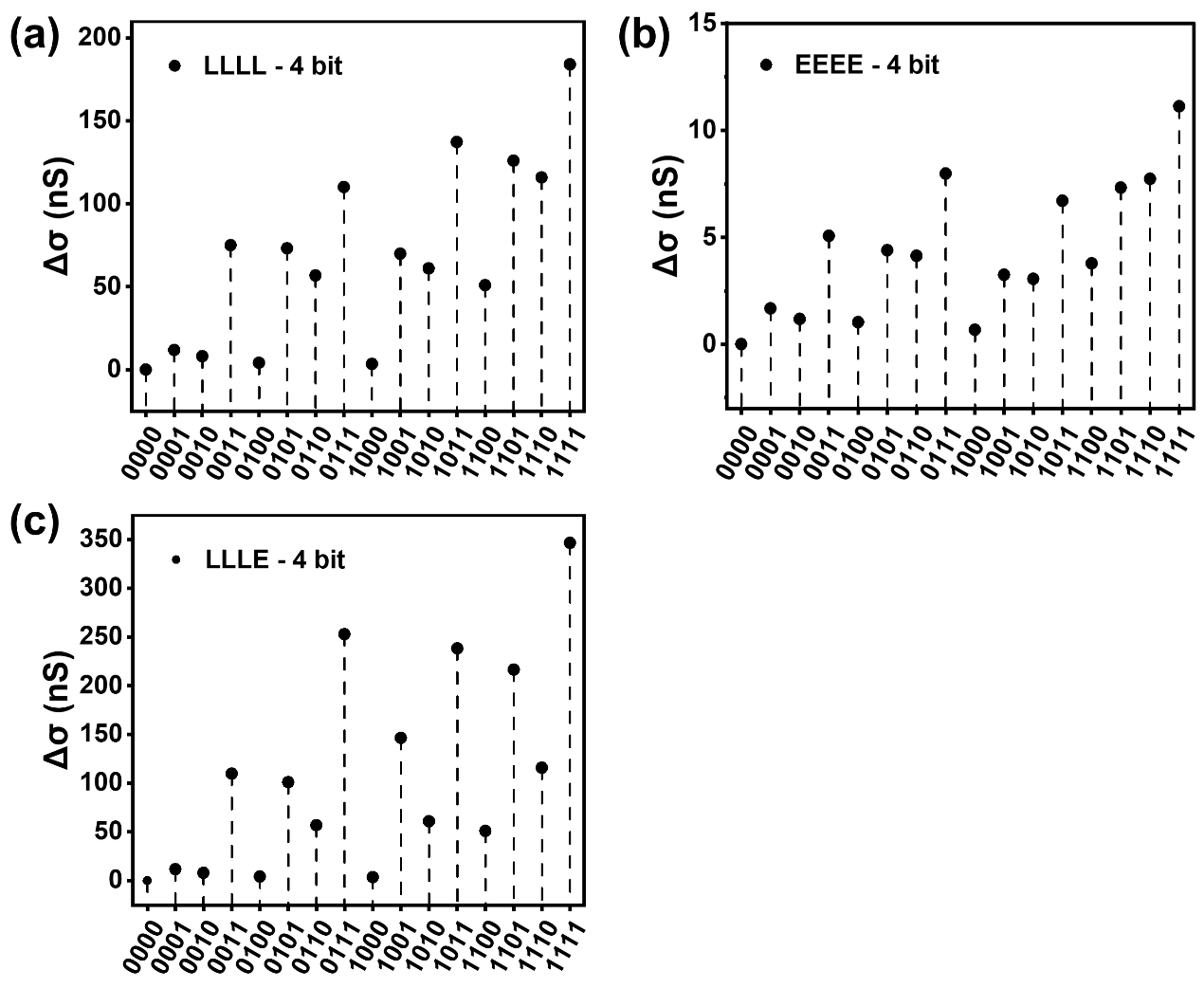


**Supplementary Figure 14.** Channel conductance response generated by 16 four-bit pulse sequences—ranging from (0000) to (1111)—under three different modes: (a) “LLLL”, (b) “EEEE”, and (c) “LLLE”. Here,"L" represents the light pulse with an intensity of 0.375 mW cm⁻² and a pulse width of 150 ms. "E" represents the electrical pulse with a gate voltage of –3.75 V and a pulse width of 150 ms. The read bias is kept at 0.1 V.


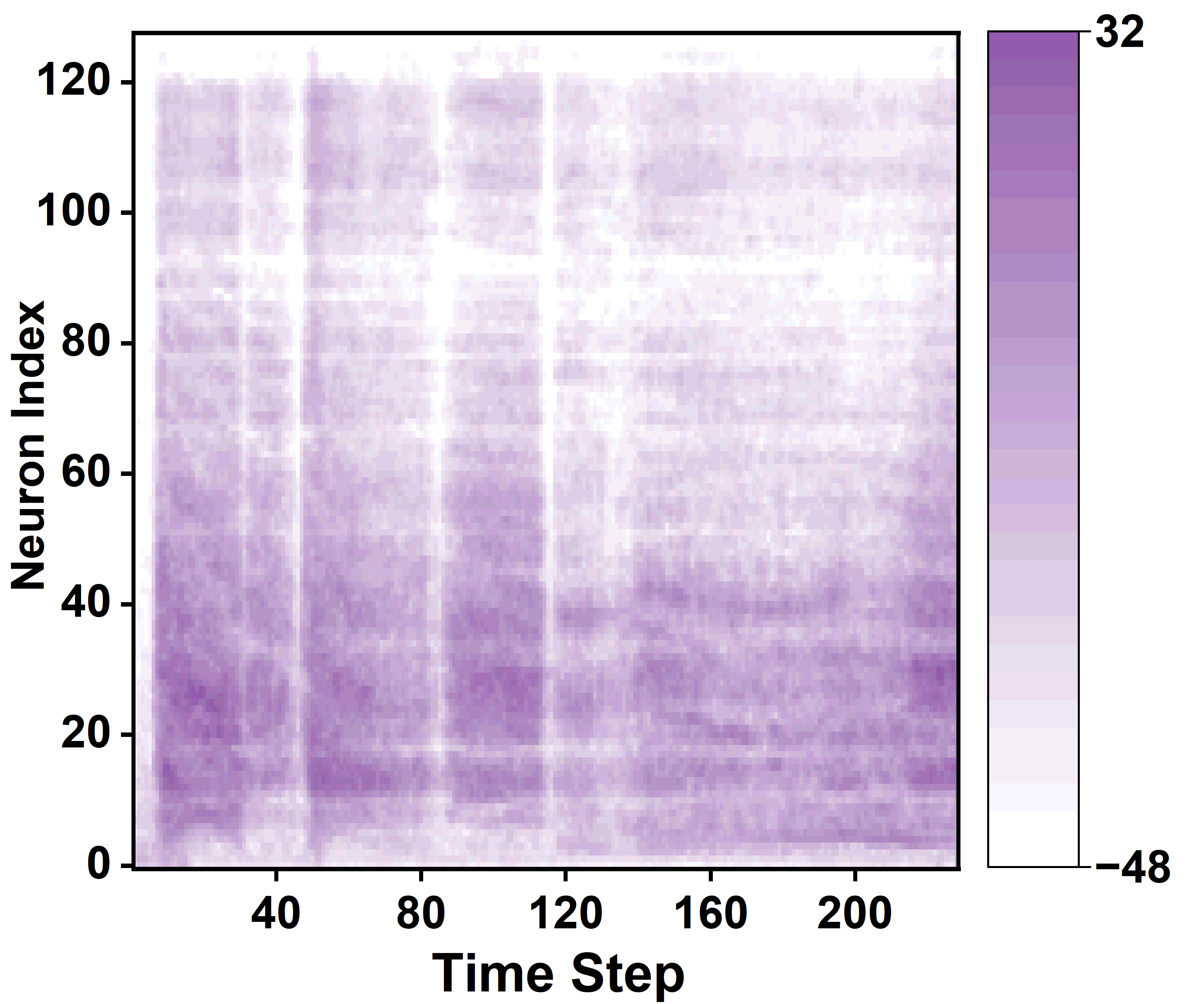


**Supplementary Figure 15.** A Mel spectrogram obtained from the power spectrum using a Mel filter bank.


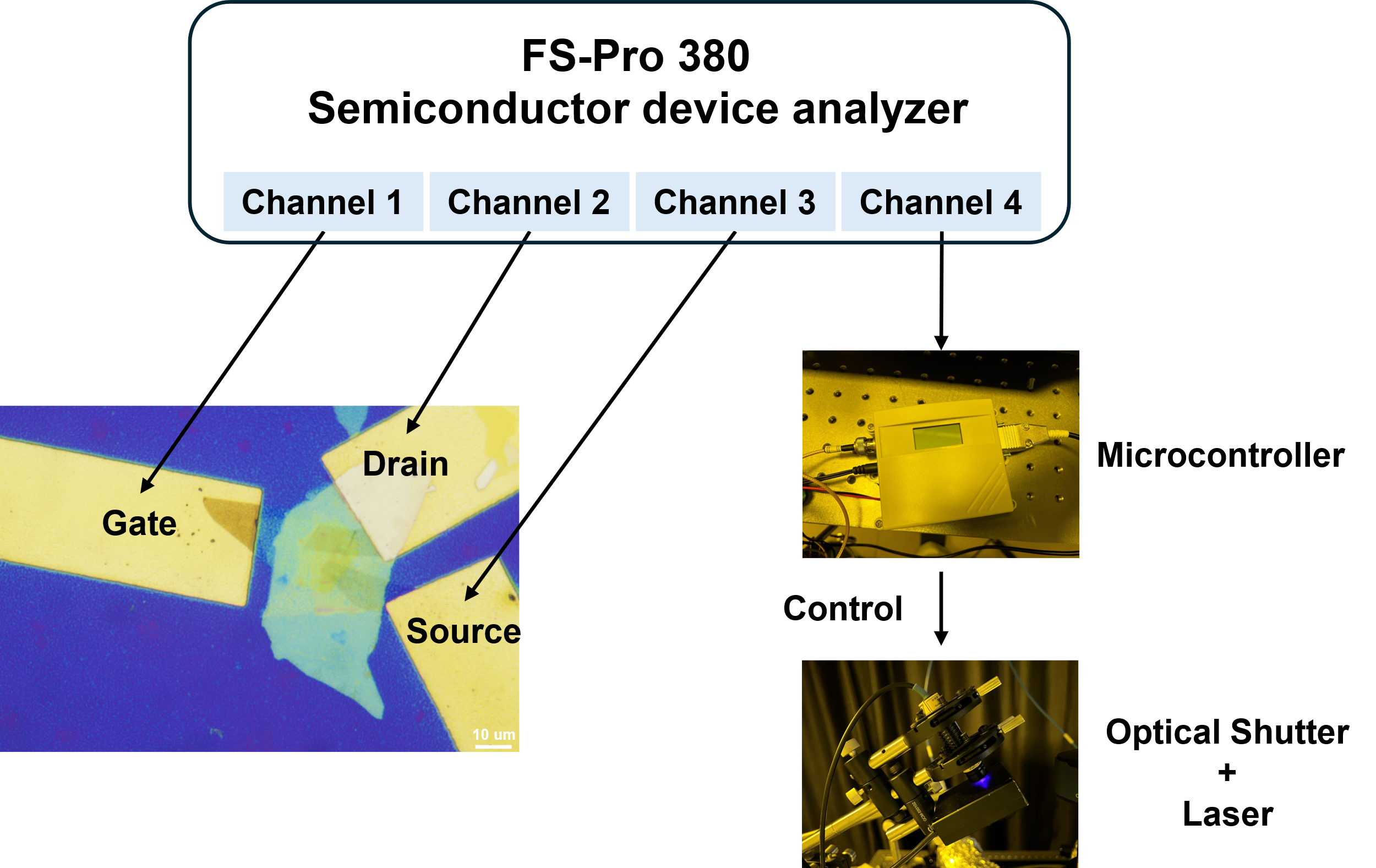


**Supplementary Figure 16.** Schematics of synergistic hybrid programming using electrical and light pulses.

**Supplementary Table 1.** The advantages and disadvantages of the common monitoring methods^[1]^

|  | **Advantages** | **Disadvantages** |
| --- | --- | --- |
| **Tagging and tracking^[2]^** | Precise movement path and range of activity | Difficulty species statistics,  Costly |
| **Drones^[3]^** | Flexibility,  Real-time | Limited battery life |
| **Remote sensing^[4]^** | Large-scale monitoring of habitat changes and animal community distribution. | Low resolution,  High cost, Obvious weather impact |
| **Camera traps^[5-6]^** | Continuous monitoring,  Rich data | Large amount of data,  Obvious weather impact |
| **Acoustic traps^[7]^** | Larger coverage area | Complex data analysis, Large environmental noise interference |
| **0D-2D FGPT** | Anti-environmental interference,  Signal complementation,  Continuous monitoring,  Larger coverage area | Relatively complex device manufacturing |

**References**

[1] A. Prosekov, A. Kuznetsov, A. Rada, S. Ivanova, *Forests* **2020**, *11*, 808.

[2] M. A. Hindell, R. R. Reisinger, Y. Ropert-Coudert, L. A. Huckstadt, P. N. Trathan, H. Bornemann, J. B. Charrassin, S. L. Chown, D. P. Costa, B. Danis, M. A. Lea, D. Thompson, L. G. Torres, A. P. Van de Putte, R. Alderman, V. Andrews-Goff, B. Arthur, G. Ballard, J. Bengtson, M. N. Bester, A. S. Blix, L. Boehme, C. A. Bost, P. Boveng, J. Cleeland, R. Constantine, S. Corney, R. J. M. Crawford, L. Dalla Rosa, P. J. N. de Bruyn, K. Delord, S. Descamps, M. Double, L. Emmerson, M. Fedak, A. Friedlaender, N. Gales, M. E. Goebel, K. T. Goetz, C. Guinet, S. D. Goldsworthy, R. Harcourt, J. T. Hinke, K. Jerosch, A. Kato, K. R. Kerry, R. Kirkwood, G. L. Kooyman, K. M. Kovacs, K. Lawton, A. D. Lowther, C. Lydersen, P. O. Lyver, A. B. Makhado, M. E. I. Marquez, B. I. McDonald, C. R. McMahon, M. Muelbert, D. Nachtsheim, K. W. Nicholls, E. S. Nordoy, S. Olmastroni, R. A. Phillips, P. Pistorius, J. Plotz, K. Putz, N. Ratcliffe, P. G. Ryan, M. Santos, C. Southwell, I. Staniland, A. Takahashi, A. Tarroux, W. Trivelpiece, E. Wakefield, H. Weimerskirch, B. Wienecke, J. C. Xavier, S. Wotherspoon, I. D. Jonsen, B. Raymond, *Nature* **2020**, *580*, 87.

[3] J. M. Robinson, P. A. Harrison, S. Mavoa, M. F. Breed, *Methods Ecol. Evol.* **2022**, *13*, 1899.

[4] J. Cavender-Bares, F. D. Schneider, M. J. Santos, A. Armstrong, A. Carnaval, K. M. Dahlin, L. Fatoyinbo, G. C. Hurtt, D. Schimel, P. A. Townsend, S. L. Ustin, Z. H. Wang, A. M. Wilson, *Nat. Ecol. Evol.* **2022**, *6*, 506.

[5] N. Sundaram, S. D. Meena, *Artificial Intelligence Review* **2023**, *56*, 1.

[6] C. Chen, J. F. Brodie, R. Kays, T. J. Davies, R. Liu, J. T. Fisher, J. Ahumada, W. McShea, D. Sheil, B. Agwanda, M. H. Andrianarisoa, R. D. Appleton, R. Bitariho, S. Espinosa, M. M. Grigione, K. M. Helgen, A. Hubbard, C. M. Hurtado, P. A. Jansen, X. Jiang, A. Jones, E. L. Kalies, C. Kiebou‐Opepa, X. Li, M. G. M. Lima, E. Meyer, A. B. Miller, T. Murphy, R. Piana, R. C. Quan, C. T. Rota, F. Rovero, F. Santos, S. Schuttler, A. Uduman, J. K. van Bommel, H. Young, A. C. Burton, *Conserv. Lett.* **2022**, *15*, e12865.

[7] T. Bradfer-Lawrence, N. Bunnefeld, N. Gardner, S. G. Willis, D. H. Dent, *Ecol. Indicators* **2020**, *115*, 106400.
